# Evaluating the feasibility of study methods for a future trial-based economic evaluation of a multistage shared decision-making program for type 2 diabetes mellitus: Protocol for a cluster-randomized controlled pilot study

**DOI:** 10.1371/journal.pone.0300944

**Published:** 2025-08-05

**Authors:** Anna Tichler, Dorijn F.L. Hertroijs, Ghislaine A.P.G. van Mastrigt, Martijn C.G.J. Brouwers, Dirk Ruwaard, Arianne M. J. Elissen

**Affiliations:** 1 Department of Health Services Research, Care and Public Health Research Institute (CAPHRI), Faculty of Health, Medicine and Life Sciences (FHML), Maastricht University, Maastricht, the Netherlands; 2 Care and Public Health Research Institute (CAPHRI), Faculty of Health, Medicine and Life Sciences (FHML), Maastricht University, Maastricht, the Netherlands; 3 Department of Internal Medicine, Division of Endocrinology and Metabolic Disease, Maastricht University Medical Center+, Maastricht, the Netherlands; Murcia University, Spain, SPAIN

## Abstract

**Introduction:**

We developed a multistage shared decision-making program for type 2 diabetes that aims to support person-centered type 2 diabetes management in primary care. The program consists of an online patient decision aid, a preparatory consult for patients, and interprofessional training for healthcare professionals. The short- and long-term effectiveness of the multistage shared decision-making program needs to be researched in a trial-based economic evaluation. To evaluate the feasibility of study methods for such an evaluation, we will conduct a pilot study. In this article, we outline its protocol, which focuses on sample recruitment and retention, study management, and feasibility of outcome and cost measurements.

**Methods and analysis:**

The multistage shared decision-making program will be pilot-tested in a cluster-randomized controlled trial in four primary care practices (located in the region of Gorinchem, the Netherlands) using a convergent parallel mixed-methods approach. The intervention practices will adopt the program, whereas the control practices provide usual care. Data collection will include recruitment, retention, and consent rates, patients’ sociodemographic and clinical characteristics, and the assessment of primary and secondary outcomes of the future trial-based economic evaluation. We will also collect data on the usage behavior of patients when completing questionnaires of the primary and secondary outcomes (i.e., time needed to complete questionnaires). Semi-structured interviews with patients will be conducted to obtain insights into the understandability and usability of measurement tools. Moreover, focus groups with healthcare professionals from participating practices will be organized to complement the quantitative data on sample representativeness and to assess the study management challenges of participating practices.

**Discussion:**

The pilot will address uncertainties around the feasibility of a future trial-based economic evaluation, focusing on sample recruitment and retention, study management, and the feasibility of outcome and cost measurements. The results will guide the improvement of study procedures for the economic evaluation of our multistage shared decision-making program for type 2 diabetes.

**Trial registration:**

The study is registered at ClinicalTrials.gov: NCT06410768.

## Introduction

Type 2 diabetes mellitus (T2DM) is associated with significant levels of morbidity and mortality, reduced quality of life, and increased healthcare costs [1]. National and international clinical guidelines for T2DM emphasize the need for person-centered care, including shared decision-making (SDM), to decide on the best treatment course for an individual patient [2–4]. SDM is complex in T2DM care because of the availability of many pharmacological and lifestyle treatment options. Patients and healthcare professionals (HCPs) face difficult trade-offs in aligning treatment attributes (e.g., efficacy, side effects) with patients’ clinical factors and preferences. SDM support is essential to benefit fully from person-centered T2DM care. We developed a multistage SDM program for T2DM that combines (1) an online patient decision aid (PDA) with (2) a preparatory consult for patients, and (3) interprofessional training in the PDA and SDM for HCPs [5]. The program was co-created with patients with T2DM, healthcare professionals involved in T2DM care, and patient organizations.

Evidence shows that PDAs, when used across various health conditions, can reduce patients’ decisional conflict, help them become more informed, increase involvement and satisfaction with treatment choices, and enhance communication between patients and HCPs [6]. Some evidence suggests that PDAs, through their effective support of SDM, can lead to improvements in treatment adherence and persistence, resulting in better health outcomes and cost reduction. However, the available evidence remains limited [6]. Our multistage SDM program for T2DM is a complex intervention as defined by the Medical Research Council (MRC) [7]. It consists of multiple, interacting components, targets a diverse group of end-users, and influences a range of short- and long-term outcome measures. To estimate short- and long-term effectiveness, a trial-based economic evaluation with the multistage SDM program needs to be conducted. This evaluation will strengthen the limited evidence on the impact of person-centered care and SDM on mid and long-term outcomes such as treatment adherence, health outcomes, and costs.

Previous randomized controlled trials researching the effects of SDM support through PDAs for T2DM experienced several challenges related to study procedures (e.g., recruitment), resources (e.g., time necessary to complete questionnaires), and study management (e.g., personnel and data management for participating practices) [8–12]. These challenges include difficulties in recruiting patients, understandability of questionnaires, timely recruitment, and inadvertent recruitment bias. Small-scale piloting is therefore crucial to address uncertainties around the feasibility of study methods and to improve the study procedures of a trial-based economic evaluation [12,13]. This article outlines the protocol for a cluster-randomized controlled pilot study of a multistage SDM program, including a PDA for T2DM in the Netherlands, compared to usual care. The pilot study is designed to assess the feasibility of conducting a future trial-based economic evaluation. The pilot study specifically focuses on sample recruitment and retention, study management, and feasibility of outcomes and cost measurements. A mixed-methods approach will be used to identify and address potential challenges for future trial-based economic evaluations.

### Research questions

The pilot aims to address uncertainties regarding the feasibility of study methods. This includes researching strategies to tackle recruitment and retention challenges, ensuring a representative and diverse sample of patients with T2DM for the trial-based economic evaluation [14]. Moreover, acknowledging the high workload and time constraints experienced by HCPs, we aim to gain insights into how to minimize burdens on participating primary care practices [15,16]. Finally, considering the complexity of the measurement process and the understandability of the measurement tools, the outcomes and costs will be measured in the pilot study to refine study procedures for the trial-based economic evaluation. Therefore, the research questions of this pilot study are:

1. What strategies can be employed to effectively recruit and retain a demographically and clinically diverse sample of patients with T2DM?
2. How can we support primary care practices in effectively managing the challenges associated with study participation?
3. How can we feasibly measure relevant SDM outcomes, treatment adherence, health outcomes, and costs from a societal perspective for T2DM using valid and reliable measurement instruments?

## Methods and analysis

**Fig 1:**
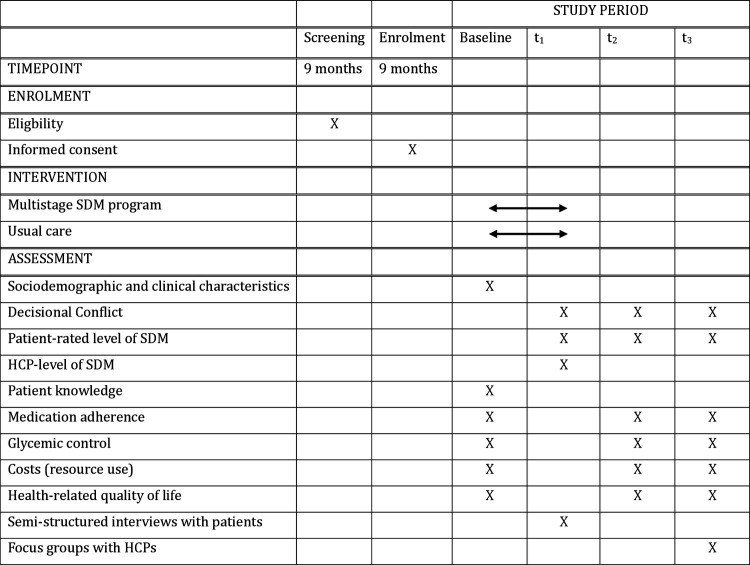
SPIRIT schedule of enrollment, intervention, and assessment. t1 = directly following the clinical encounter where a treatment decision is made; t2 = 3-month follow-up; t3 = 9-month follow-up. SDM: shared decision-making, HCPs: healthcare professionals.

This protocol follows a combination of the Consolidated Standards of Reporting Trials (CONSORT) extension to pilot trials [17] and the Standard Protocol Items: Recommendations for Interventional Trials (SPIRIT) checklist for reporting protocol studies [18,19], as described by Lehana Thabane [20] (Supplementary Files 1 and 2). For conducting and reporting the future trial-based economic evaluation, the Dutch guidelines for economic evaluation [21] and the Consolidated Health Economic Evaluation Reporting Standards 2022 (CHEERS 2022) Statement [22] will be followed, respectively. The pilot study is planned to run for 18 months, from early November 2023 to late April 2025. Patient recruitment and data collection are scheduled to take place from February 2024 to January 2025. This will be followed by data analysis and reporting. Fig 1 illustrates the SPIRIT schedule for the study [18,19]. The study is registered at ClinicalTrials.gov (NCT06410768).

### Setting

The majority of patients with T2DM in the Netherlands (90% in 2022) are treated in a primary care setting organized by care groups [23]. Care groups are collaborations between HCPs (general practitioners and affiliated personnel) and are responsible for organizing, coordinating, and providing care for patients with T2DM in their region [24]. A team comprising of a general practitioner and a practice/diabetes nurse provides treatment following the national guidelines for T2DM of the Dutch College of General Practitioners (NHG) [25]. Since most Dutch patients with T2DM are treated in primary care, the multistage SDM program was developed based on the NHG guidelines for T2DM and will be pilot-tested in a general practice setting. We collaborate with the primary care group ‘Huisarts & Zorg’, a group of 75 general practices located in the region of Gorinchem (a municipality in South Holland) to recruit practices and patients. Four primary care practices from the care group ‘Huisarts & Zorg’ will be included in this pilot study.

### Study design

The multistage SDM program will be piloted in a cluster-randomized controlled trial using a convergent parallel mixed-methods approach to answer questions related to sample recruitment and retention, study management, and feasibility of outcome and cost measurements. Randomization will be conducted by cluster (i.e., primary care practices) to avoid possible contamination between the intervention and control group [26]. Two primary care practices will be randomly assigned to the intervention group and two to the control group. Simple randomization will be used to assign each primary care practice to a group with an equal probability (1:1 allocation) using a computerized random number generator [27]. Patients with T2DM and HCPs are not blinded to their assigned group. Patients and HCPs from the intervention practices will have access to the multistage SDM program. They will receive an account to gain access to the PatientPlus platform, where the PDA is available. Patients and HCPs from the control practices will provide and receive usual care according to the NHG guidelines for T2DM [25]. They will not have access to the multistage program. Data will be collected from patients and HCPs in both the intervention and control practices.

### Participants

To be able to recruit a diverse population of patients with T2DM, we aim to include general practices from the care group ‘Huisarts & Zorg’ that differ in terms of the sociodemographic background of their patient panels. HCPs from the participating practices will be asked to recruit patients who: 1) are eighteen years or older; 2) need to decide on T2DM treatment based on the NHG guideline (medication and/or lifestyle); and 3) speak Dutch at a necessary level to complete questionnaires and ensure involvement in SDM. We will only exclude patients with severe cognitive impairments that hamper SDM. Patients will be enrolled in the study after receiving face-to-face and written information about the study from their HCP and after giving written informed consent.

### Intervention

**Fig 2:**
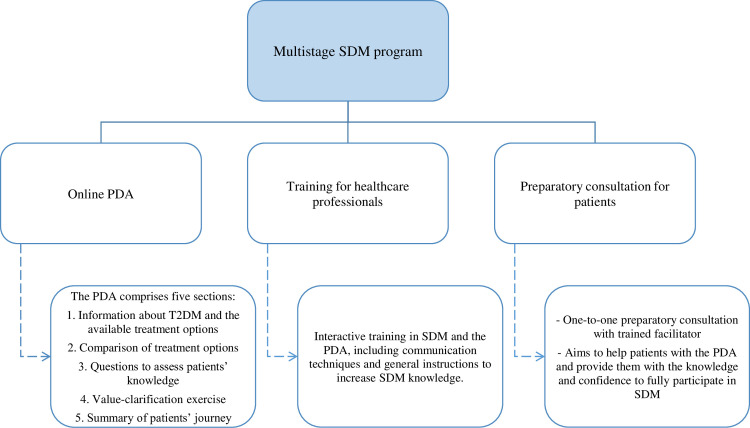
Overview of the multistage shared decision-making program for type 2 diabetes mellitus. SDM: shared decision-making; PDA: patient decision aid; T2DM: Type 2 Diabetes Mellitus.

The multistage SDM program combines an online PDA with a preparatory consultation for patients as well as an interprofessional training in the PDA and SDM for HCPs (Fig 2). The program was co-created with a multidisciplinary steering group representing all relevant stakeholders in Dutch diabetes care. The development of the PDA for T2DM is described in detail elsewhere [5].

The PDA is available in the online catalog of PatientPlus (https://www.keuzehulp.info/front-page/keuzehulpen/diabetes-type-2, Dutch only), the largest supplier of PDAs in the Netherlands. In line with the International Patient Decision Aids Standards (IPDAS) guidance, the PDA comprises of five sections: 1) information about T2DM and the available treatment options; 2) a comparison of treatment options based on, for example, the risk of cardiovascular disease and effect on daily life; 3) questions to assess patients’ knowledge; 4) value-clarification exercise; and 5) summary of the patient’s journey [28,29].

To prepare and empower patients for SDM, our multistage program comprises a one-to-one preparatory consultation with a trained facilitator. Each practice can decide whether a practice nurse, medical assistant or other HCP will serve as a trained facilitator. The consultation will last 20–30 minutes and is intended to help patients effectively use the PDA and provide them with the knowledge and confidence needed to fully participate in SDM. The preparatory consultation takes place before the clinical encounter where the treatment decision is made by a patient and HCP (i.e., a general practitioner or specialized nurse who can prescribe medication).

All participating HCPs (including the trained facilitators) from included intervention practices receive a 2-hour interactive interprofessional training in SDM and the PDA, including communication techniques and general instructions to increase SDM knowledge. HCPs will receive accreditation for their participation in the training. The training is offered by PatientPlus. One intervision meeting between the trained facilitators from all participating practices will be held to stimulate interprofessional training.

### Sample size calculation

The pilot study is a preparation for a large trial-based economic evaluation and therefore setting the sample size for the pilot study in order to minimize the total sample size of the pilot study and main trial together is the most suitable method of sample size calculation [30]. The sample size calculation for the pilot study is based on a 90% powered main trial and an estimated medium (between 0.3 and 0.7) effect size in the decisional conflict score (primary outcome of the trial-based economic evaluation) [31]. The decisional conflict score was chosen as the primary outcome as it captures the patient’s uncertainty and quality of the decision-making process, which aligns with the outcomes of the multistage SDM program [32]. Moreover, it is a validated questionnaire that is widely used in PDA-supported SDM research. Using the stepped rules of thumb, the sample size for the pilot study would be 30 patients with T2DM [30]. Due to possible loss to follow-up and drop-out, the sample size will be increased by a third. Thus, the sample size for this pilot study will be set at 40 patients with T2DM, with 20 patients assigned to each arm.

### Outcome measures

The pilot study focuses on three aspects: 1) sample recruitment and retention; 2) study management; and 3) feasibility of outcome and cost measurements. Each aspect has its own relevant outcome measures and measurement instruments.

#### Research question 1. Sample recruitment and retention.

To assess the extent to which a representative sample of T2DM patients is included and retained, quantitative data will be collected on: (1) recruitment, retention and consent rates; (2) time required to recruit the target sample size; and (3) sociodemographic and clinical characteristics. To interpret the quantitative data and learn how to improve sample representativeness for the trial-based economic evaluation, additional qualitative data will be collected through one-hour focus groups with HCPs from the participating practices.

#### Research question 2. Study management.

The focus groups with HCPs from participating practices will also be used to assess practices’ study management challenges. An interview guide, consisting of a set of semi-structured questions related to study management (e.g., did the practice have the time to perform the tasks they committed to doing? Did they experience any capacity issues?) will be used to guide the focus group.

#### Research question 3. Feasibility of outcome and cost measurements.

We will assess the feasibility of primary and secondary outcome measurements of the future trial-based economic evaluation. The primary and secondary outcomes will solely be measured to assess its measurement feasibility and not to determine the (cost-) effectiveness and cost-utility of the multistage SDM program. The primary outcomes focuses on short-term SDM outcomes. Primary outcomes include patient decisional conflict (using the 16-item Decisional Conflict Scale, DCS [32]), level of SDM as perceived by patients (based on the 3-item CollaboRATE survey [33,34] and SDM-Q-9 questionnaire [35]), level of SDM as perceived by HCPs (using the SDM-Q-Doc questionnaire [35]), and patient knowledge (with 9 tailor-made questions assessing patient’s understanding of the glucose-lowering treatments). The DCS, CollaboRATE, SDM-Q-9, and SDM-Q-Doc questionnaires will be used due to their validity and wide applicability in assessing SDM in healthcare. The long-term cost-effectiveness and cost-utility of our multistage SDM program will serve as secondary outcomes in the trial-based economic evaluation. To compare the multistage program with usual care and estimate the costs of improving individual’s quality of life, it is essential to assess societal costs and health-related quality of life for conducting a cost-utility analysis [36]. In the future trial-based economic evaluation, we will also conduct a cost-effectiveness analysis to estimate the costs of achieving improvements in relevant health outcomes (i.e., glycemic control). Therefore, secondary outcomes include glycemic control (HbA1c obtained via the HCP), societal costs (measured with an adapted version of the iMTA Productivity Costs Questionnaire (iPCQ) and iMTA Medical Consumption Questionnaire (iMCQ) [37,38]), health-related quality of life (measured with the Dutch EuroQol (EQ) 5D-5L questionnaire assessing quality of life [39]), and medication adherence (measured with the Medication Adherence Report Scale [40] and prescription data obtained via the pharmacist).

Table 1 provides an overview of the primary and secondary outcomes of the future trial-based economic evaluation, including their measurement tools. We will conduct semi-structured interviews with patients to gain insights into the understandability and accessibility of the measurement tools. Interviews will be conducted either in person or online via Microsoft Teams, depending on the patients’ preference. Participation in the interviews is optional for patients. They can indicate their consent to be contacted for an interview by selecting the appropriate option in the informed consent form. A topic list will be developed to guide the interviews. Moreover, we will collect data on the usage behavior of patients when completing the questionnaires (i.e., time needed to complete questionnaires) and the amount of missing data. The focus groups with HCPs from participating practices will also be used to evaluate the understandability of the SDM-Q-Doc questionnaire.

**Table 1:** Outcomes of the future large-scale trial-based economic evaluation, including their measurement tools.

Outcome	Measurement tool	Components
**Patients**		
Decisional Conflict	Decisional Conflict Scale (DCS) [32]	Patients will be asked to reflect on the treatment decision they made with their HCP and respond to 16 statements in the DCS using a five-point Likert scale (from completely agree to completely disagree).
Level of SDM	CollaboRATE survey [33,34]	3 statements that assess patients’ perception of being informed and engaged in decision-making steps on a scale of zero (no effort was made) to nine (every effort was made)
	SDM-Q-9 questionnaire [35]	The questionnaire contains nine statements each describing a different step of the SDM process. All items are scored on a six-point Likert scale from zero (completely disagree) to five (completely agree). The questionnaire also includes two open-ended questions: one about the health problem addressed during the consultation and the other about the decision made.
Patient knowledge	9 tailor-made multiple-choice questions	Assessing patients’ understanding of the (risks and benefits of) glucose-lowering treatments.
Medication adherence	Medication Adherence Report Scale (MARS) [40]	Assessing intentional and unintentional non-adherence (self-reported). Patients are asked to rate the frequency of events (i.e., forgetting a dose, changing a dose) on a five-point Likert Scale (ranging from never to always).
	Prescription data	Patients’ pharmacy records for all T2DM medication will be collected.
Glycemic control	Primary care data	Glycemic control is assessed by obtaining HbA1c data from primary care practice.
Costs (resource use)	An adapted version of the iMCQ and iPCQ questionnaire [37,38]	The adapted version of the iMTA Productivity Costs Questionnaire (iPCQ) and the iMTA Medical Consumption Questionnaire (iMCQ) aims to measure relevant (non-)healthcare use and costs that are significant to patients and their families.
Health-related quality of life	Dutch EuroQol (EQ) 5D-5L (quality of life) [39]	The EQ 5D-5L assesses quality of life and includes the EQ-5D dimension and the EQ visual analog scale (VAS). EQ-5D comprises five dimensions: mobility, self-care, usual activities, pain, and anxiety. Each dimension of the EQ-5D is scored on a five-point Likert score (from no problems to extreme problems). The EQ VAS is used to assess the patient’s self-reported health on a visual analog scale.
**Healthcare professionals**		
Level of SDM	SDM-Q-Doc questionnaire [35]	SDM-Q-9 questionnaire adapted to the HCP viewpoint to assess the extent of SDM during a consultation from the HCP perspective.

HCP: healthcare professional; SDM: shared decision-making.

### Data collection and timeline

The pilot study is planned to run for 18 months, from early November 2023 to late April 2025. Patient recruitment and data collection are scheduled to take place from February 2024 to January 2025. This will be followed by data analysis and reporting. Patients from the intervention practices are requested to complete questionnaires via the online PatientPlus platform and patients from the control practices will complete questionnaires via Qualtrics [41]. They will receive automatic email reminders to complete the follow-up questionnaires at the 3- and 9-month follow-up. Additionally, automated reminders will be sent if the patient has not yet completed the questionnaire. HCPs from the control and intervention practices will complete the questionnaire via Qualtrics [41]. The schedule of study enrolment and assessment can be found in Fig 1. Baseline measurements will capture sociodemographic and clinical patient characteristics, patient knowledge, medication adherence, costs, and health-related quality of life. In both study arms, patients complete the DCS, CollaboRATE survey, and SDM-Q-9 questionnaire directly following the clinical encounter where a treatment decision is made. At the same time, HCPs complete the SDM-Q-Doc questionnaire. Follow-ups at 3 and 9 months will facilitate the measurement of medication adherence, costs, and health-related quality of life. Semi-structured interviews with patients will be held within one month of study participation and the focus groups with HCPs from participating practices will be held at the end of the 9-month implementation period. After completing data collection at the 9-month follow-up, we will gather information on the glycemic control of participating patients by obtaining their HbA1c values at baseline, and 3- and 9-month follow-ups, through their primary care practice.

### Data analysis

Descriptive statistics will be presented as counts and percentages for categorical variables. For continuous variables, mean and standard deviation will be reported for normally distributed data. If the data is not normally distributed, the median and interquartile range (IQR) will be reported instead. The variables include sociodemographic and clinical characteristics, outcomes of the future trial-based economic evaluation, and estimates related to feasibility (e.g., recruitment rates and time to complete questionnaires). Moreover, we will analyze the missing data by identifying the amount of missing data and complementing this information with the results of the qualitative analysis. This involves cross-referencing the amount of missing data with patient interview results, where insights into the understandability of the questionnaires are gathered. Additionally, during focus groups with healthcare professionals, possible factors influencing incomplete questionnaires or patient dropout will be discussed. Descriptive analysis will be performed in Rstudio [42]. Due to the nature of the pilot study, no statistical tests on the primary and secondary outcomes will be conducted.

Interviews and focus groups will be audio-recorded, transcribed ad verbatim, and anonymized to protect participant’s confidentiality. The data will subsequently be analyzed in ATLAS.ti using thematic analysis with an inductive approach following three steps [43,44]. First, the transcripts will be read and re-read in a process called ‘familiarization’ to gain an in-depth understanding of the data. Second, phrases, sentences, and paragraphs with meaningful topics will be extracted and labeled with a code for each interview transcript, with this process conducted independently by two researchers. An initial codebook will be developed based on the interview guide, and codes will be added and adjusted as they emerge from the data. The coded transcripts from both researchers will be compared, and discrepancies will be discussed to promote reflexivity. Inter-coder reliability will be established and maintained throughout the coding process. Third, themes are developed by clustering codes with similar meanings or interrelations, to understand, interpret, and report the main insights flowing from the data. The analysis of the interviews and focus groups will be used to improve the questionnaires (e.g., question formulation and possible adaptations to the cost questionnaire), recruitment strategies, and study procedures.

The data management process (i.e., data collection, data processing, data quality, and data analysis) and possible improvements thereof will be discussed with the research team in three 2-hour meetings throughout the study period.

### Data monitoring and quality control

The data will be internally monitored by a member of the research team, ensuring accuracy in data entry and completeness to maintain data integrity. The research team will also track patient enrollment and recruitment rates across the participating general practices, as well as monitor consent forms to ensure ethical compliance. To ensure correct integration of the PDA into clinical practice, participating HCPs from the intervention practices will receive training in SDM and using the PDA. Throughout the study, the research team will maintain regular contact (via phone or email every three months) with the general practices to gather continuous feedback on recruitment and implementation of the program. Additionally, an intervision meeting with HCPs from the intervention practices will be held to address potential challenges and to collect data on how each practice is implementing the multistage SDM program.

### Ethical considerations and declarations

The Medical Ethics Review Committee of the academic hospital of Maastricht (azM) and Maastricht University confirmed that the Medical Research Involving Human Subjects Act does not apply to the pilot study and that official approval is not required (METC2023−0114). See Supplementary File 3 for the full protocol approved by the Medical Ethics Review Committee. Patients will be enrolled in the study after receiving face-to-face and written information about the research and after giving written informed consent. Participants’ data will be used and retained by the researchers of Maastricht University in compliance with the EU General Data Protection Regulation. Some of the data will be collected via PatientPlus. PatientPlus has an information security management system that is ISO27001 and NEN7510 certified. Researchers will be provided with an account for PatientPlus to access the research data of patients. PatientPlus has only access to the anonymized data. The collected data will be stored on the protected server of Maastricht University. All participants in this study will be given an ID number to ensure their confidentiality. Data in reports and publications of this pilot study will not be traceable to the research participants. Research participants have the right to withdraw from the research and withdraw their consent to the use of personal data at any time during the study. The data from the pilot study will be available upon request.

## Discussion

This protocol outlines the approach for pilot testing our multistage SDM program in primary care. Our primary objective is to address uncertainties around the feasibility of study methods, focusing on aspects such as sample recruitment and retention, study management, and the feasibility of outcome and cost measurements. Given that T2DM affects a large and diverse group of patients, it is important to ensure that the study participants accurately represent this diversity, as it is essential for our trial-based economic evaluation [14]. Previous randomized controlled trials researching the effects of PDAs for T2DM experienced recruitment challenges [8–11]. These trials reported difficulties in recruiting sufficient participants, timely recruitment, and inadvertent recruitment bias. Moreover, some trials were unable to include a representative sample of patients with T2DM [9,11]. It is also important to acknowledge that the PDA is in a digital format, and questionnaires need to be completed electronically. This may pose a limitation for individuals with low digital literacy, especially among older adults [45–47]. Therefore, we place a strong emphasis on the feasibility of the study processes in terms of recruitment, retention, and consent rates. This will improve our understanding of strategies to include a representative and diverse group of participants, and we can apply these insights to the recruitment process of the trial-based economic evaluation.

HCPs in primary care practices are faced with a high workload, time constraints, and stress which are identified as barriers to research participation [15,16,48]. To avoid willingness and capacity problems of the participating practices related to the study, it is important to limit time expenses and paperwork, and to provide adequate information and support. Therefore, as part of the pilot study, we focus on how we can support general practices in effectively managing the challenges associated with study participation. These insights will be instrumental in ensuring the successful implementation of the multistage SDM program into routine practice while minimizing the burden on practices.

Valid and reliable instruments are available (except for the cost questionnaire) for the primary and secondary outcomes of the trial-based economic evaluation [32–35,39,40]. The measurement process can be complex because outcomes are collected using different methods, from different sources, and at various time points. It is important to avoid data management problems and ensure successful data triangulation in the large-scale study. Hence, the primary and secondary outcomes will be measured and analyzed during the small-scale pilot study. We also focus on the understandability and accessibility of the measurement tools and possible improvements thereof. This is especially important since approximately 24.5% of the Dutch population experience low health literacy and may therefore face difficulties when completing questionnaires [49]. Overall, the insights gained from the pilot study will guide the refinement of the study procedures and intervention components for the trial-based economic evaluation.

The proposed pilot study has some possible limitations. Given the nature of the intervention, both patients and HCPs are not blinded to their assigned group. HCPs, who are responsible for delivering the intervention, and patients with T2DM will be aware of whether they are receiving the intervention. This may influence how patients report outcomes, such as their perception of the decision-making process, which may not be directly related to the intervention. Additionally, HCPs may unintentionally treat patients differently or alter their behavior during consultations, leading to differences in outcomes that are not directly related to the intervention itself. All HCPs in the intervention practices will receive standardized training to ensure consistent use of the PDA and reduce the risk of variability in how the intervention is implemented. However, the intervention offers some flexibility, allowing HCPs to adapt the program and easily integrate it into their daily practice. Moreover, by grouping general practices rather than individuals in a cluster-randomized design, we reduce the likelihood of cross-contamination between the two groups. Another limitation might be the possible exclusion of patients with limited (digital) health literacy, since the PDA and all included questionnaires are digital. As described earlier, with this pilot study we aim to improve recruitment strategies to ensure a representative sample in the trial-based economic evaluation.

Our pilot study will contribute to the existing knowledge of effectively implementing PDAs into clinical practice. Previous research showed that only 44% of existing PDAs for various conditions are effectively integrated into clinical practice following their trial [46]. The trial-based economic evaluation strengthens the limited evidence on the impact of person-centered care and SDM on mid and long-term outcomes such as treatment adherence, health outcomes, and societal costs. It will contribute to gaining a more comprehensive understanding of the potential benefits and challenges of integrating person-centered SDM interventions into practice. Addressing these knowledge gaps is essential to convince HCPs, policymakers, and payers to invest in the widespread implementation of effective SDM support for person-centered care.

### Dissemination plan

The results of this pilot study will be disseminated via conference presentations and international peer-reviewed scientific journals. Moreover, attention will be given to promoting the multistage SDM program among patients with T2DM and HCPs within primary care. This will help the future recruitment of patients with T2DM and primary care practices for the trial-based economic evaluation. Our collaboration with the steering group, consisting of patients with T2DM, patient organizations, and HCPs, established at the beginning of the development of the multistage SDM program, will be continued. The steering group and our collaboration with care group ‘Huisarts & Zorg’ and PatientPlus add valuable expertise and experience from practice and policy as future end-users of our multistage SDM program. This collaboration will improve the implementation and dissemination of the program.

## Supporting information

S1 File SPIRIT checklist. (DOCX)

S2 File CONSORT checklist. (DOC)

S3 File Protocol. (DOCX)

## References

[1] IDF Diabetes Atlas. Brussels, Belgium: International Diabetes Federation. 2021. https://www.diabetesatlas.org

[2] American Diabetes Association Professional Practice Committee. 9. pharmacologic approaches to glycemic treatment: standards of care in diabetes-2024. Diabetes Care. 2024;47(Suppl 1):S158–78. doi: 10.2337/dc24-S009 38078590 PMC10725810

[3] Davies MJ, Aroda VR, Collins BS, Gabbay RA, Green J, Maruthur NM, et al. Management of Hyperglycemia in type 2 diabetes, 2022. a consensus report by the American Diabetes Association (ADA) and the European Association for the Study of Diabetes (EASD). Diabetes Care. 2022;45(11):2753–86. doi: 10.2337/dci22-0034 36148880 PMC10008140

[4] Nederlands Huisartsen Genootschap. NHG-standaard diabetes mellitus type 2 (versie 5.6). 2023.

[5] Tichler A, Hertroijs DFL, Ruwaard D, Brouwers MCGJ, Elissen AMJ. Development of a patient decision aid for type 2 diabetes mellitus: a patient-centered approach. BMC Prim Care. 2025;26(1):81. doi: 10.1186/s12875-025-02772-7 40121397 PMC11929313

[6] Stacey D, Lewis KB, Smith M, Carley M, Volk R, Douglas EE, et al. Decision aids for people facing health treatment or screening decisions. Cochrane Database Syst Rev. 2024;2024(1). doi: 10.1002/14651858.CD001431.pub6 PMC10823577 38284415

[7] Skivington K, Matthews L, Simpson SA, Craig P, Baird J, Blazeby JM, et al. A new framework for developing and evaluating complex interventions: update of Medical Research Council guidance. BMJ. 2021;374:n2061. doi: 10.1136/bmj.n2061 34593508 PMC8482308

[8] Branda ME, LeBlanc A, Shah ND, Tiedje K, Ruud K, Van Houten H, et al. Shared decision making for patients with type 2 diabetes: a randomized trial in primary care. BMC Health Serv Res. 2013;13:301. doi: 10.1186/1472-6963-13-301 23927490 PMC3751736

[9] Kellar I, Mann E, Kinmonth AL, Prevost AT, Sutton S, Marteau TM. Can informed choice invitations lead to inequities in intentions to make lifestyle changes among participants in a primary care diabetes screening programme? Evidence from a randomized trial. Public Health. 2011;125(9):645–52. doi: 10.1016/j.puhe.2011.05.010 21764087

[10] Mathers N, Ng CJ, Campbell MJ, Colwell B, Brown I, Bradley A. Clinical effectiveness of a patient decision aid to improve decision quality and glycaemic control in people with diabetes making treatment choices: a cluster randomised controlled trial (PANDAs) in general practice. BMJ Open. 2012;2(6):e001469. doi: 10.1136/bmjopen-2012-001469 23129571 PMC3532975

[11] Mullan RJ, Montori VM, Shah ND, Christianson TJH, Bryant SC, Guyatt GH, et al. The diabetes mellitus medication choice decision aid: a randomized trial. Arch Intern Med. 2009;169(17):1560–8. doi: 10.1001/archinternmed.2009.293 19786674

[12] Thabane L, Ma J, Chu R, Cheng J, Ismaila A, Rios LP, et al. A tutorial on pilot studies: the what, why and how. BMC Med Res Methodol. 2010;10:1. doi: 10.1186/1471-2288-10-1 20053272 PMC2824145

[13] van Teijlingen E, Hundley V. The importance of pilot studies. Nurs Stand. 2002;16(40):33–6. doi: 10.7748/ns2002.06.16.40.33.c3214 12216297

[14] Black SA. Diabetes, diversity, and disparity: what do we do with the evidence? Am J Public Health. 2002;92(4):543–8. doi: 10.2105/ajph.92.4.543 11919048 PMC1447113

[15] McKinley N, McCain RS, Convie L, Clarke M, Dempster M, Campbell WJ, et al. Resilience, burnout and coping mechanisms in UK doctors: a cross-sectional study. BMJ Open. 2020;10(1):e031765. doi: 10.1136/bmjopen-2019-031765 31988223 PMC7045750

[16] West CP, Dyrbye LN, Shanafelt TD. Physician burnout: contributors, consequences and solutions. J Intern Med. 2018;283(6):516–29. doi: 10.1111/joim.12752 29505159

[17] Eldridge SM, Chan CL, Campbell MJ, Bond CM, Hopewell S, Thabane L, et al. CONSORT 2010 statement: extension to randomised pilot and feasibility trials. Pilot Feasibility Stud. 2016;2:64. doi: 10.1186/s40814-016-0105-8 27965879 PMC5154046

[18] Chan A-W, Tetzlaff JM, Altman DG, Laupacis A, Gøtzsche PC, Krleža-Jerić K, et al. SPIRIT 2013 statement: defining standard protocol items for clinical trials. Ann Intern Med. 2013;158(3):200–7. doi: 10.7326/0003-4819-158-3-201302050-00583 23295957 PMC5114123

[19] Chan A-W, Tetzlaff JM, Gøtzsche PC, Altman DG, Mann H, Berlin JA, et al. SPIRIT 2013 explanation and elaboration: guidance for protocols of clinical trials. BMJ. 2013;346:e7586. doi: 10.1136/bmj.e7586 23303884 PMC3541470

[20] Thabane L, Lancaster G. A guide to the reporting of protocols of pilot and feasibility trials. Pilot Feasibility Stud. 2019;5:37. doi: 10.1186/s40814-019-0423-8 30858987 PMC6393983

[21] Zorginstituut Nederland. Richtlijn voor het uitvoeren van economische evaluaties in de gezondheidszorg. 2024: 38.

[22] Husereau D, Drummond M, Augustovski F, de Bekker-Grob E, Briggs AH, Carswell C, et al. Consolidated Health Economic Evaluation Reporting Standards 2022 (CHEERS 2022) statement: updated reporting guidance for health economic evaluations. Value Health. 2022;25(1):3–9. doi: 10.1016/j.jval.2021.11.1351 35031096

[23] InEen. Benchmark transparante ketenzorg 2022. Rapportage zorgprogramma’s diabetes mellitus type 2, COPD, astma en vasculair risico management bij patiënten met een hartvaatziekte en een verhoogd risico op hartvaatziekten. 2023.

[24] Struijs JN, van Til JT, Baan CA. Experimenteren met de keten-dbc diabetes. Bilthoven: RIVM. 2009.

[25] Nederlands Huisartsen Genootschap (NHG). Behandeling bij diabetes type 2. Available from: https://www.thuisarts.nl/diabetes-type-2/ik-heb-diabetes-type-2#behandeling-bij-diabetes-type-2. 2022.

[26] Fayers PM, Jordhøy MS, Kaasa S. Cluster-randomized trials. Palliat Med. 2002;16(1):69–70. doi: 10.1191/0269216302pm503xx 11963457

[27] Kang M, Ragan BG, Park J-H. Issues in outcomes research: an overview of randomization techniques for clinical trials. J Athl Train. 2008;43(2):215–21. doi: 10.4085/1062-6050-43.2.215 18345348 PMC2267325

[28] Elwyn G, O’Connor A, Stacey D, Volk R, Edwards A, Coulter A, et al. Developing a quality criteria framework for patient decision aids: online international Delphi consensus process. BMJ. 2006;333(7565):417. doi: 10.1136/bmj.38926.629329.AE 16908462 PMC1553508

[29] Elwyn G, O’Connor AM, Bennett C, Newcombe RG, Politi M, Durand M-A, et al. Assessing the quality of decision support technologies using the International Patient Decision Aid Standards instrument (IPDASi). PLoS One. 2009;4(3):e4705. doi: 10.1371/journal.pone.0004705 19259269 PMC2649534

[30] Whitehead AL, Julious SA, Cooper CL, Campbell MJ. Estimating the sample size for a pilot randomised trial to minimise the overall trial sample size for the external pilot and main trial for a continuous outcome variable. Stat Methods Med Res. 2016;25(3):1057–73. doi: 10.1177/0962280215588241 26092476 PMC4876429

[31] Yu CH, Ivers NM, Stacey D, Rezmovitz J, Telner D, Thorpe K, et al. Impact of an interprofessional shared decision-making and goal-setting decision aid for patients with diabetes on decisional conflict--study protocol for a randomized controlled trial. Trials. 2015;16:286. doi: 10.1186/s13063-015-0797-8 26116444 PMC4486130

[32] O’Connor AM. Validation of a decisional conflict scale. Med Decis Making. 1995;15(1):25–30. doi: 10.1177/0272989X9501500105 7898294

[33] Barr PJ, Thompson R, Walsh T, Grande SW, Ozanne EM, Elwyn G. The psychometric properties of CollaboRATE: a fast and frugal patient-reported measure of the shared decision-making process. J Med Internet Res. 2014;16(1):e2. doi: 10.2196/jmir.3085 24389354 PMC3906697

[34] Elwyn G, Barr PJ, Grande SW, Thompson R, Walsh T, Ozanne EM. Developing CollaboRATE: a fast and frugal patient-reported measure of shared decision making in clinical encounters. Patient Educ Couns. 2013;93(1):102–7. doi: 10.1016/j.pec.2013.05.009 23768763

[35] Rodenburg-Vandenbussche S, Pieterse AH, Kroonenberg PM, Scholl I, van der Weijden T, Luyten GPM, et al. Dutch translation and psychometric testing of the 9-item Shared Decision Making Questionnaire (SDM-Q-9) and Shared Decision Making Questionnaire-Physician Version (SDM-Q-Doc) in primary and secondary care. PLoS One. 2015;10(7):e0132158. doi: 10.1371/journal.pone.0132158 26151946 PMC4494856

[36] Fox-Rushby JC. Economic Evaluation. London: Oxford University Press. 2006.

[37] iMTA Productivity and Health Research Group. Handleiding iMTA Medical Cost Questionnaire (*i*MCQ). Rotterdam: iMTA, Erasmus Universiteit Rotterdam 2018. Available from: www.imta.nl.

[38] iMTA Productivity and Health Research Group. Handleiding iMTA Productivity Cost Questionnaire (iPCQ). iMTA, Erasmus Universiteit. 2018. www.imta.nl

[39] EuroQol. EuroQol EQ-5D. Available from: https://euroqol.org/eq-5d-instruments/eq-5d-3l-about/. 2017.

[40] Chan AHY, Horne R, Hankins M, Chisari C. The Medication Adherence Report Scale: A measurement tool for eliciting patients’ reports of nonadherence. Br J Clin Pharmacol. 2020;86(7):1281–8. doi: 10.1111/bcp.14193 31823381 PMC7319010

[41] Qualtrics. Provo, Utah, USA. 2005. Available from: https://www.qualtrics.com/.

[42] Team R. RStudio: Integrated Development for R. 1.4.1106 ed bbbbbbbb . Boston, MA: RStudio, PBC. 2020.

[43] ATLAS.ti Scientific Software Development GmbH. ATLAS.ti Scientific Software Development GmbH.

[44] Braun V, Clarke V. Using thematic analysis in psychology. Qualitative Research in Psychology. 2006;3(2):77–101. doi: 10.1191/1478088706qp063oa

[45] Hargittai E, Piper AM, Morris MR. From internet access to internet skills: digital inequality among older adults. Univ Access Inf Soc. 2018;18(4):881–90. doi: 10.1007/s10209-018-0617-5

[46] Stacey D, Suwalska V, Boland L, Lewis KB, Presseau J, Thomson R. Are patient decision aids used in clinical practice after rigorous evaluation? a survey of trial authors. Med Decis Making. 2019;39(7):805–15. doi: 10.1177/0272989X19868193 31423911

[47] van Deursen AJ, van Dijk JA. The first-level digital divide shifts from inequalities in physical access to inequalities in material access. New Media Soc. 2019;21(2):354–75. doi: 10.1177/1461444818797082 30886536 PMC6380454

[48] Brodaty H, Gibson LH, Waine ML, Shell AM, Lilian R, Pond CD. Research in general practice: a survey of incentives and disincentives for research participation. Ment Health Fam Med. 2013;10(3):163–73. 24427184 PMC3822664

[49] Willems AEM, Heijmans M, Brabers AEM, Rademakers J. Gezondheidsvaardigheden in Nederland: factsheet cijfers 2021. Utrecht: Nivel. 2022.

