# Supplementary material for: Evaluating the feasibility of study methods for a future trial-based economic evaluation of a multistage shared decision-making program for type 2 diabetes mellitus: Protocol for a cluster-randomized controlled pilot study
Source: PLoS One. 2025 Aug 5;20(8):e0300944. doi: 10.1371/journal.pone.0300944 (PMC12324081; doi:10.1371/journal.pone.0300944)
Supplement: S3 File — (DOCX) [file pone.0300944.s003.docx]

**RESEARCH PROTOCOL**

**Date: 30-11-2023**

**Version: 1**

**PROTOCOL TITLE** ‘Evaluating the feasibility of a future trial-based economic evaluation of a multistage shared decision-making program for the treatment of type 2 diabetes mellitus: a pilot study’

| **Protocol ID** | NL85395.068.23 |
| --- | --- |
| **Short title** | Economic evaluation of a multistage shared decision-making program for type 2 diabetes: a pilot study |
| **EudraCT number** | Not applicable |
| **Version** | 1 |
| **Date** | 30-11-2023 |
| **Principal investigator(s) (in Dutch: hoofdonderzoeker/ uitvoerder)** | Arianne Elissen |
| **Other investigators** | Dirk Ruwaard  Martijn Brouwers  Dorijn Hertroijs  Anna Tichler |
| **Sponsor (in Dutch: verrichter/opdrachtgever)** | Maastricht University |
| **Subsidising party** | ZonMw, the Netherlands Organisation for Health Research and Development  070 349 5465 |
| **Independent expert (s)** | Rowan Smeets |
| **Laboratory sites** | Not applicable |
| **Pharmacy** | Not applicable |

**PROTOCOL SIGNATURE SHEET**

| **Name** | **Signature** | **Date** |
| --- | --- | --- |
| **Head of Department:**  Sandra Zwakhalen |  |  |
| **Coordinating Investigator/Project Leader/Principal Investigator:**  Arianne Elissen |  |  |

**TABLE OF CONTENTS**

1. INTRODUCTION AND RATIONALE 8

2. OBJECTIVES 10

3. STUDY DESIGN 10

4. STUDY POPULATION 11

4.1 Population (base) 11

4.2 Inclusion criteria 12

4.3 Exclusion criteria 12

4.4 Sample size calculation 12

5. TREATMENT OF SUBJECTS 13

5.1 Investigational product/treatment 13

5.2 Use of co-intervention (if applicable) 18

5.3 Escape medication (if applicable) 18

6. INVESTIGATIONAL PRODUCT 18

6.1 Name and description of investigational product(s) 18

6.2 Summary of findings from non-clinical studies 18

6.3 Summary of findings from clinical studies 18

6.4 Summary of known and potential risks and benefits 18

6.5 Description and justification of route of administration and dosage 18

6.6 Dosages, dosage modifications and method of administration 18

6.7 Preparation and labelling of Investigational Medicinal Product 18

6.8 Drug accountability 18

7. NON-INVESTIGATIONAL PRODUCT 18

7.1 Name and description of non-investigational product(s) 19

7.2 Summary of findings from non-clinical studies 19

7.3 Summary of findings from clinical studies 19

7.4 Summary of known and potential risks and benefits 19

7.5 Description and justification of route of administration and dosage 19

7.6 Dosages, dosage modifications and method of administration 19

7.7 Preparation and labelling of Non Investigational Medicinal Product 19

7.8 Drug accountability 19

8. METHODS 19

8.1 Study parameters/endpoints 19

8.1.1 Main study parameter/endpoint 19

8.1.2 Secondary study parameters/endpoints (if applicable) 20

8.1.3 Other study parameters (if applicable) 20

8.2 Randomisation, blinding and treatment allocation 20

8.3 Study procedures 20

8.4 Withdrawal of individual subjects 26

8.4.1 Specific criteria for withdrawal (if applicable) 26

8.5 Replacement of individual subjects after withdrawal 26

8.6 Follow-up of subjects withdrawn from treatment 26

8.7 Premature termination of the study 26

9. SAFETY REPORTING 26

9.1 Temporary halt for reasons of subject safety 26

9.2 AEs, SAEs and SUSARs 26

9.2.1 Adverse events (AEs) 26

9.2.2 Serious adverse events (SAEs) 27

9.2.3 Suspected unexpected serious adverse reactions (SUSARs) 27

9.3 Annual safety report 27

9.4 Follow-up of adverse events 27

9.5 Data Safety Monitoring Board (DSMB) / Safety Committee 27

10. STATISTICAL ANALYSIS 28

10.1 Primary study parameter(s) 28

10.2 Secondary study parameter(s) 28

10.3 Other study parameters 29

10.4 Interim analysis (if applicable) 29

11. ETHICAL CONSIDERATIONS 29

11.1 Regulation statement 29

11.2 Recruitment and consent 29

11.3 Objection by minors or incapacitated subjects (if applicable) 29

11.4 Benefits and risks assessment, group relatedness 29

11.5 Compensation for injury 31

11.6 Incentives (if applicable) 31

12. ADMINISTRATIVE ASPECTS, MONITORING AND PUBLICATION 31

12.1 Handling and storage of data and documents 31

12.2 Monitoring and Quality Assurance 33

12.3 Amendments 33

12.4 Annual progress report 33

12.5 Temporary halt and (prematurely) end of study report 33

12.6 Public disclosure and publication policy 34

13. STRUCTURED RISK ANALYSIS 34

13.1 Potential issues of concern 34

13.2 Synthesis 34

14. REFERENCES 34

**LIST OF ABBREVIATIONS AND RELEVANT DEFINITIONS**

| **ADA** | **American Diabetes Association** |
| --- | --- |
| **AE** | **Adverse Event** |
| **AR** | **Adverse Reaction** |
| **CCMO** | **Central Committee on Research Involving Human Subjects; in Dutch: Centrale Commissie Mensgebonden Onderzoek** |
| **DCS** | **Decisional Conflict Scale** |
| **CV** | **Curriculum Vitae** |
| **EASD** | **European Association for the Study of Diabetes** |
| **EQ-5D** | **EuroQol – 5 dimension** |
| **EQ-VAS** | **EuroQol – visual analog scale** |
| **GLP-1** | **Glucagon-like peptide 1** |
| **GP** | **General Practitioner** |
| **HCP** | **Health Care Professionals** |
| **IC** | **Informed Consent** |
| **IPDAS** | **International Patient Decision Aids Standards** |
| **METC** | **Medical research ethics committee (MREC); in Dutch: medisch-ethische toetsingscommissie (METC)** |
| **NHG** | **Dutch College of General Practitioners; in Dutch: Nederlands Huisartsen Genootschap** |
| **PDA** | **Patient Decision Aid** |
| **PIF** | **Subject Information Sheet; in Dutch: patiënteninformatie** |
| **(S)AE** | **(Serious) Adverse Event** |
| **SDM** | **Shared decision-making** |
| **SGLT-2** | **Sodium-glucose contransport-2** |
| **Sponsor** | **The sponsor is the party that commissions the organisation or performance of the research, for example, a pharmaceutical**  **company, academic hospital, scientific organisation, or investigator. A party that provides funding for a study but does not commission it is not regarded as the sponsor but referred to as a subsidising party.** |
| **SUSAR** | **Suspected Unexpected Serious Adverse Reaction** |
| **T2DM** | **Type 2 Diabetes Mellitus** |
| **WMO** | **Medical Research Involving Human Subjects Act; in Dutch: Wet Medisch-wetenschappelijk Onderzoek met Mensen** |

**SUMMARY**

**Rationale:** Previous randomized controlled trials researching the effects of shared decision-making (SDM) support through patient decision aids (PDAs) for type 2 diabetes mellitus (T2DM) experienced several challenges related to study procedures, resources, and study management. These challenges include difficulties in recruiting participants, understandability of questionnaires, timely recruitment, and inadvertent recruitment bias. Small-scale piloting is crucial to address uncertainties around the feasibility of trial methods and to refine the study procedures of a large-scale economic evaluation. This protocol outlines a pilot study aimed at evaluating the feasibility of a future trial-based economic evaluation of a multistage SDM program, including a PDA for T2DM in the Netherlands. By conducting a pilot study, we aim to improve the quality of the intended trial-based economic evaluation.

**Objectives**: We will conduct a pilot study focused on the following questions to prepare for an intended economic evaluation of our multistage SDM program for T2DM: 1) How can we recruit and retain a representative sample of patients with T2DM in terms of sociodemographic and clinical background?; 2) How can we support primary care practices in managing the challenges associated with study participation?; 3) How can we feasibly measure relevant outcomes of SDM for T2DM using valid and reliable measurement instruments?

**Study design:** Our multistage SDM program will be piloted in a cluster-randomized controlled trial using a mixed-methods approach. Four primary care practices will be included, of which two will be randomly assigned to the intervention group and two to the control group.

**Study population:** Participants include adult patients with T2DM who need to decide on T2DM treatment based on the NHG guideline together with their primary care HCPs.

**Intervention**: Our multistage SDM program consists of 1) an online PDA; 2) preparatory consult to provide patients with the knowledge and confidence to participate in SDM; and 3) training to improve HCPs skills for SDM. Control practices will provide usual care.

**Main study parameters/endpoints:** The primary endpoints of the study are centered around the three objectives: 1) sample recruitment and retention (i.e. recruitment, retention, and consent rates, time required to target, and sociodemographic and clinical characteristics); 2) study management (time constraints and capacity issues); and 3) feasibility of outcome measurements (understandability and accessibility of measurement tools of the short- and long-term outcomes of the future economic evaluation).

**Nature and extent of the burden and risks associated with participation, benefit and group relatedness:** Participants will be asked to complete questionnaires. No medication interventions, treatments, or invasive procedures are involved, thereby minimizing physical strain and medical risks for the participants. We do not expect that there are any risks associated with completing the questionnaires. Moreover, the study requires limited involvement of patients and therefore imposes no burden.

1. INTRODUCTION AND RATIONALE

Diabetes mellitus is a highly prevalent disease that affects 10.5% of the world’s adult population, of which over 90% have type 2 diabetes mellitus (T2DM) [1]. T2DM is associated with significant levels of morbidity and mortality, reduced quality of life, and increased healthcare costs [2]. National and international clinical guidelines for T2DM emphasize the need for person-centered care, including shared decision-making (SDM), to decide on the best treatment course for an individual patient [3-5]. Care should be respectful of and responsive to individual patient preferences, needs and values, and ensure that these guide clinical decisions [6]. However, with many pharmacological and lifestyle treatment options available, SDM is complex in T2DM care. Patients and healthcare professionals (HCPs) face difficult trade-offs in aligning treatment attributes (e.g. efficacy, side effects) with patients’ clinical factors and preferences. SDM support is needed to reap the full benefits of person-centered T2DM care.

In general, patient decision aids (PDAs) are a type of SDM support that aims to help patients and HCPs make informed decisions [2]. PDAs include information about the treatment options that require consideration, along with their associated risks and benefits. Evidence shows that PDAs can reduce patients’ decisional conflict, make them better informed, more involved, and satisfied with their treatment choices, and have a positive effect on the communication between patients and HCPs [7]. There is some evidence suggesting that PDAs, through their effective support of SDM, can lead to improvements in treatment adherence and persistence, thereby resulting in better health outcomes and cost reduction [7]. However, the available evidence for these effects remains limited [7]. While the potential benefits of using PDAs in healthcare are promising, research shows that only 44% of PDAs are used in practice following their trial [8]. This highlights the need for implementation research for PDAs. Economic evaluation strengthens the limited evidence about the impact of person-centered care and SDM on mid and long-term outcomes such as treatment adherence, health outcomes, and costs [7].

Previous randomized controlled trials researching the effects of SDM support through PDAs for T2DM experienced several challenges related to study procedures (i.e. recruitment, understandability of questionnaires), resources (i.e. time and resource problems), and study management (i.e. human and data management problems) [9-12]. These challenges include, for example, difficulties in recruiting participants, understandability of questionnaires, timely recruitment, and inadvertent recruitment bias. Recognizing the challenges faced in previous research, small-scale piloting is crucial to address uncertainties around the feasibility of trial methods and to refine the study procedures of a large-scale economic evaluation [13, 14]. This research protocol outlines the protocol for a pilot study aimed at evaluating the feasibility of a future trial-based economic evaluation of a multistage SDM program, including a PDA for T2DM in the Netherlands, compared to usual care [15]. By conducting a pilot study, we aim to improve the quality of the intended trial-based economic evaluation. The pilot study specifically focuses on sample recruitment and retention, study management, and feasibility of outcome measurement, aiming to identify and address potential challenges in the intended large-scale economic evaluation. The reasoning for why we specifically focus on these three topics is described below.

Given that T2DM affects a large and diverse group of patients, it is important to ensure that the study participants accurately represent this diversity, as it is essential for our intended economic evaluation [16]. Previous randomized controlled trials researching the effects of PDAs for T2DM experienced recruitment challenges [9-12]. These trials reported difficulties in recruiting sufficient participants, timely recruitment, and inadvertent recruitment bias. Moreover, some trials were unable to include a representative sample of patients with T2DM [10, 12]. We will therefore place a strong emphasis on the feasibility of the study processes in terms of recruitment, retention, and consent rates within the pilot study. This will improve our understanding and we can apply these insights to the recruitment process of the intended economic evaluation.

HCPs in primary care practices are faced with a high workload, time constraints, and stress which are also identified as barriers to research participation [17-19]. To avoid willingness and capacity problems of the participating practices related to the study, it is important to limit time expenses and paperwork, and to provide adequate information and support. Therefore, as part of the pilot study, we focus on how we can support general practices in effectively managing the challenges associated with study participation. These insights will be instrumental in ensuring the successful implementation of the multistage program into routine practice while minimizing the additional burden on practices.

There are valid and reliable instruments available for the primary and secondary outcomes of the intended trial-based economic evaluation. The measurement process can be complex since outcomes are collected using different methods, from different sources, and at various time points. It is important to avoid data management problems and ensure successful data triangulation in the large-scale study. Hence, the primary and secondary outcomes will be measured and analyzed during the small-scale pilot study. In the pilot study, we also focus on the understandability and accessibility of the measurement tools and possible improvements thereof. This is especially important since approximately 24.5% of the Dutch population experience low health literacy and may therefore face difficulties when completing questionnaires [20]. Overall, the insights gained from the pilot study will guide the refinement of the study procedures and intervention components for the intended economic evaluation.

1. OBJECTIVES

Primary Objective:

Small-scale piloting is crucial to address uncertainties around the feasibility of trial procedures, resources, and study management [13, 14]. Our pilot aims to improve the conduct and quality of an intended trial-based economic evaluation of our multistage SDM program for T2DM. The pilot study will specifically focus on strategies to deal with recruitment and retention challenges and gain insights into how to minimize additional burdens on participating primary care practices. Moreover, the outcomes of the intended large-scale economic evaluation will be measured to identify possible data management issues and subsequently refine study procedures. We will conduct a pilot study focused on the following questions to prepare for an intended economic evaluation of our multistage SDM program for T2DM:

1. How can we recruit and retain a representative sample of patients with T2DM in terms of sociodemographic and clinical background?
2. How can we support primary care practices in managing the challenges associated with study participation?
3. How can we feasibly measure relevant outcomes of SDM for T2DM using valid and reliable measurement instruments?
4. STUDY DESIGN

This protocol follows the Standard Protocol Items: Recommendations for Interventional Trials (SPIRIT) 2013 statement and guidelines [21, 22], and the Consolidated Health Economic Evaluation Reporting Standards 2022 (CHEERS 2022) Statement [23] (attached to the protocol as separate documents). The Dutch guidelines for economic evaluation will also be followed [24].

*Study design*

Our multistage SDM program will be piloted in a cluster-randomized controlled trial using a mixed-methods approach to answer questions related to sample recruitment and retention, study management, and feasibility of outcome measurements. Four primary care practices will be included in this pilot study. Randomization will be conducted by cluster (i.e. primary care practices) to avoid possible contamination between the intervention and control group [25]. Two primary care practices will be randomly assigned to the intervention group and two to the control group. Simple randomization will be used to assign each primary care practice to a group with an equal probability (1:1 allocation) using a computerized random number generator [26]. Given the nature of the intervention, both patients with T2DM and HCPs are not blinded to the group assigned to them. The intervention practices will have access to the multistage SDM program. Patients and HCPs in the control practices will provide and receive usual care according to the national guidelines for T2DM of the Dutch College of General Practitioners (NHG) [5]. They will not have access to the multistage program. Data will be collected from patients with T2DM and healthcare professionals from both the intervention and control practices.

*Study duration*

The study will last 18 months: 3 months for study preparation, 9 months for implementation (i.e. patient recruitment and data collection) of the SDM program, and 6 months for data analysis and reporting.

*Study setting*

The majority of patients with T2DM in the Netherlands (90% in 2022) are treated in a primary care setting organized by care groups [27]. Care groups are collaborations between healthcare professionals (general practitioners and affiliated personnel) and are responsible for organizing, coordinating, and providing care for patients with T2DM in their region [28]. A team comprising a general practitioner and practice/diabetes nurse provides treatment following the national guidelines for T2DM of the NHG. The national guidelines underwent significant revisions in 2018 and 2021 allowing the use of new pharmacological agents (e.g., sodium-glucose cotransport 2 [SGLT2] inhibitors and glucagon-like peptide 1 [GLP-1] receptor agonists) for high-risk patients [5]. Patients are considered high risk if they have, amongst others, a history of cardiovascular disease or heart failure. The SGLT2 inhibitors and GLP-1 receptor agonists are also included in the treatment guidelines of the American Diabetes Association (ADA) and the European Association for the Study of Diabetes (EASD) [4]. Since most Dutch patients with T2DM are treated in primary care, the multistage SDM program was developed based on the NHG guideline for T2DM and will be pilot-tested in a general practice setting.

1. STUDY POPULATION
   1. Population (base)

Healthcare professionals (i.e. general practitioners, practice nurses, medical assistants) from primary care practices and patients with T2DM will be included in this study. To recruit practices and patients, we collaborate with the primary care group ‘Huisarts & Zorg’, a provider network of 75 general practices in the region of Gorinchem (a municipality in South Holland). This established collaboration enhances the likelihood of successful recruitment of primary care practices for this pilot study. Four primary care practices from care group ‘Huisarts & Zorg’ will be included in this pilot study. Healthcare professionals from participating primary care practices will be asked to recruit adult patients with T2DM. Recruiting patients through their healthcare professionals is anticipated to increase the likelihood of study participation. Patients will be enrolled in the study after receiving face-to-face and written information about the research and after giving written informed consent (see section 11.2 for detailed information).

- 1. Inclusion criteria

In order to be eligible to participate in this study, a subject must meet all of the following criteria:

- Is diagnosed with T2DM
- Eighteen years or older
- Need to decide on T2DM treatment based on the NHG guideline
- Multiple treatment options (medication and/or lifestyle) are possible as judged by the HCP
- Speak Dutch at a necessary level to complete questionnaires and ensure involvement in SDM

To be able to recruit a diverse population of patients with T2DM, we will select general practices that differ in terms of the sociodemographic background of their patient panels.

- 1. Exclusion criteria

A potential subject who meets any of the following criteria will be excluded from participation in this study:

- Severe cognitive impairments that hamper SDM
  1. Sample size calculation

The pilot study is a preparation for a large trial-based economic evaluation and therefore setting the sample size for the pilot study in order to minimize the total sample size of the pilot study and main trial together is the most suitable method of sample size calculation [29]. The sample size calculation for the pilot study is based on a 90% powered main trial and an estimated medium (between 0.3 and 0.7) effect size in the decisional conflict score (primary outcome of the intended economic evaluation) [30]. Using the stepped rules of thumb, the sample size for the pilot study would be 30 patients with T2DM [29]. Due to possible loss to follow-up and drop-out, the sample size will be increased by a third. So, the sample size for this pilot study will be set at 40 patients with T2DM, with 20 patients assigned to each arm.

1. TREATMENT OF SUBJECTS

The pilot study has a primary objective of enhancing the quality and conduct of a forthcoming large-scale trial-based economic evaluation into a multistage SDM program for T2DM. Through this pilot study, we aim to address uncertainties around the feasibility of the trial methods, with a particular emphasis on sample recruitment and retention, study management, and the feasibility of outcome measurements. It is important to note that the main goal of this pilot study is not to conduct an economic evaluation of the multistage SDM program itself, but to assess the feasibility of the future trial-based economic evaluation. Within the pilot study, we will be introducing the multistage SDM program into the existing care process. Consequently, this section of the research protocol will provide information about this program.

- 1. Investigational product/treatment

We developed a multistage SDM program for T2DM that combines (1) an online PDA with (2) a preparatory consult for patients, and (3) interprofessional training in the PDA and SDM for HCPs (Figure 1). The program was co-created with a multidisciplinary steering group representing all relevant stakeholders in Dutch diabetes care (patients with T2DM, the Diabetes Association Netherlands, the Netherlands Diabetes Federation, the Dutch College of General Practitioners, and healthcare professionals from various disciplines). The PDA was developed based on insights into what patients consider important to discuss with their HCPs when making treatment choices in T2DM, which includes quality of life and clinical outcomes, amongst others [31]. Using these insights, combined with the clinical guidance of the NHG guideline, we co-created an evidence-based PDA for T2DM [15]. The PDA is available in the online catalog of PatientPlus (<https://www.keuzehulp.info/front-page/keuzehulpen/diabetes-type-2>, Dutch only), the largest supplier of PDAs in the Netherlands. In line with the International Patient Decision Aids Standards (IPDAS) guidance, the PDA comprises five sections: (1) information about T2DM and the available treatment options; (2) a comparison of treatment options based on, for example, the risk of cardiovascular disease and effect on daily life; (3) questions to assess patients’ knowledge; (4) value and preference elicitation; and (5) summary of key points [2, 32]. The content of each section is presented in Figure 2.


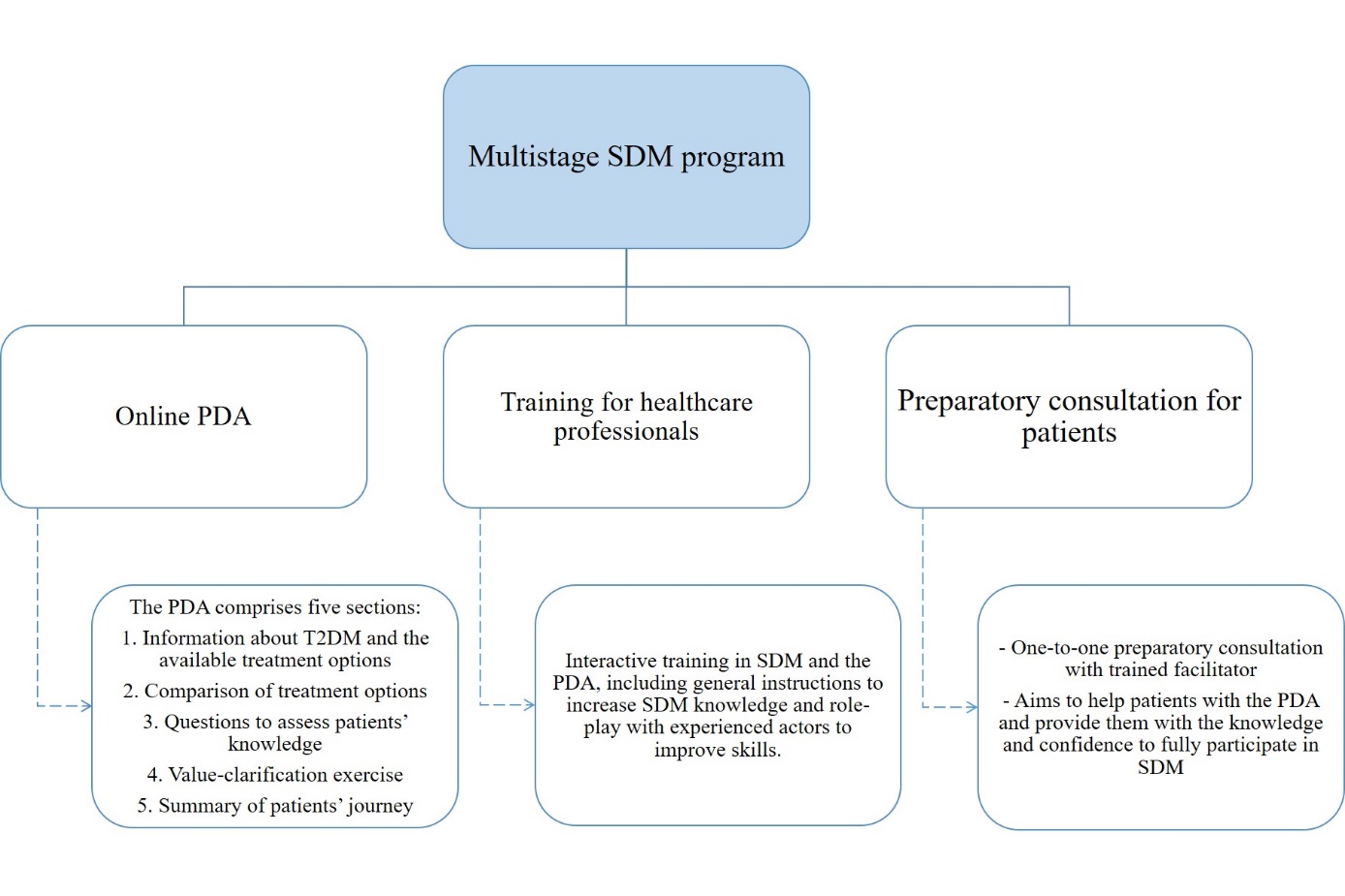


Figure 1. Overview of the multistage shared decision-making program for type 2 diabetes. SDM: shared decision-making; PDA: patient decision aid; T2DM: Type 2 Diabetes Mellitus.


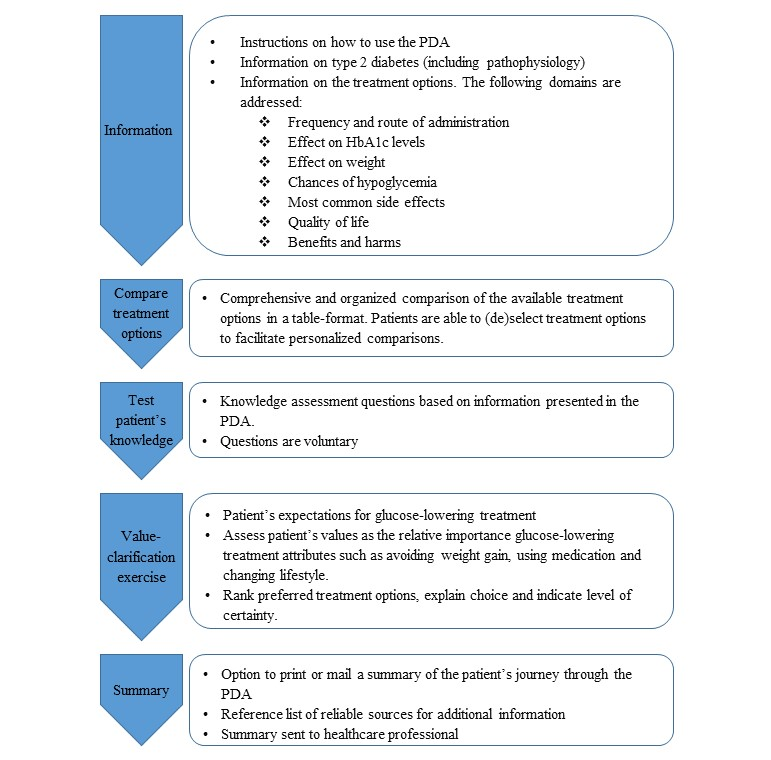


Figure 2. Content of the PDA for type 2 diabetes mellitus

The first section provides instructions on how to use the decision aid and information about T2DM, including a summarized and simplified pathophysiology of T2DM. Moreover, the different treatment options for T2DM are explained. The treatment options included in the PDA are no treatment, lifestyle changes, metformin, sulfonylureas, dipeptidyl peptidase-4 (DPP-4) inhibitors, glucagon-like peptide 1 (GLP-1) receptor agonists, sodium-glucose cotransport 2 (SGLT2) inhibitors and insulin. However, due to individual characteristics (and reimbursement restrictions), not all treatment options are suitable for every patient. Therefore, prior to the patient using the PDA, healthcare professionals select the possible treatment options for each individual in the PDA. In the first and second sections of the PDA, the patient will thus receive information only on the relevant treatment options. Nevertheless, the treatment options ‘no treatment’ and ‘lifestyle change’ will at all times be compared with other medical treatment options. It is a standard feature of all PatientPlus PDAs to include the ‘no treatment’ option, which is also a criterion in the IPDAS quality framework [2]. The ‘lifestyle change’ option is included as a standard option since patients considered the ‘lifestyle’ attribute an important conversation topic in the T2DM treatment decision-making process [31]. Furthermore, the steering group wanted to emphasize the importance of lifestyle changes in treating T2DM.

For each treatment option, information is provided on its characteristics, such as frequency and route of administration (for medical treatment options). The most important conversation topics in the T2DM treatment decision-making process, as valued by patients [31], have also been incorporated in this section of the PDA. This includes quality of life, clinical outcomes, long-term diabetes complications, and short-term adverse events of medication. Both positive and negative impact of each treatment on a patient’s quality of life is provided. For example, metformin may improve quality of life by reducing diabetes symptoms and lowering the risk of cardiovascular disease but may also have negative effects such as side effects and the need to take pills daily. The information on clinical outcomes is divided into the treatment’s effect on HbA1c levels and weight. Short-term adverse events of medication are separated into the risk of hypoglycemia and the most common side effects of each medication type. The information on long-term diabetes complications is divided into the risk of cardiovascular disease and other micro- and macrovascular complications that may occur as a consequence of T2DM. All the aforementioned information is summarized into a short list categorized into the benefits and harms of each treatment option.

The second section of the PDA presents a comprehensive and organized comparison of the available treatment options in a table format. The table presents a concise summary of each treatment, including the treatment’s characteristics (i.e., frequency and route of administration), effect on HbA1c levels and weight, long-term diabetes complications (including risk on cardiovascular disease), risk of hypoglycemia, most common side effects, and benefits and harms. The patient can select and deselect treatments and their characteristics for a personalized comparison.

The third section consists of knowledge assessment questions (N=9) based on the information presented in the PDA. These questions are included to assess whether patients correctly understood the information presented in the PDA. The questions are formatted as multiple-choice. For example, patients will be asked the following question: ‘Which medication increases the risk of hypoglycemia?’. If the patient is uncertain of the answer, they are recommended to read the relevant information in sections 1 and 2 of the PDA again or discuss it with their healthcare professional. The patient has the option to skip the questions.

The fourth section of the PDA includes a value-clarification exercise to support patients in determining their values and preferences related to their glucose-lowering treatment. This section also incorporates the attribute ‘quality of life’ and is divided into three parts. In the first part, patients are asked to share their treatment expectations. This includes describing what quality of life means to them and how they would like to see their quality of life improve during or after treatment. Second, the patient’s values and preferences are assessed using statements on several attributes, such as avoiding weight gain, using medication, changing lifestyle, and avoiding adverse events of medication. For example, patients are presented with choices such as ‘I want to lose weight’, ‘I do not mind if my weight increases’, or ‘I have no opinion’, with corresponding treatment options provided. In the third part, patients are asked to rank their preferred treatment options, explain their choice for the most preferred option, and indicate their level of certainty. Additionally, they can write down any remaining questions or concerns for their healthcare professional. Throughout the PDA, patients can take notes, which will be saved for them.

The final section enables patients to print, download, or mail a summary of the patient’s journey through the PDA, including the answers to the questions in sections three and four, and their notes. The summary can also be sent to their healthcare professional. The PDA explicitly does not provide advice for a particular treatment. It aims to help patients and healthcare professionals to decide on the most suitable treatment. In this section, patients are also able to find a list of reliable sources for additional information on T2DM and the treatment options.

The preparatory consult and interprofessional training are added to facilitate the PDA’s uptake in practice, as research shows that key implementation barriers include limited patient preparation and empowerment for SDM as well as a lack of trust, clarity, and understanding of how to use PDAs and undertake SDM among HCPs [33-36]. To prepare and empower patients for SDM, they will receive a one-to-one preparatory consultation with a trained facilitator (e.g. practice nurse or medical assistant). This consultation will last 20-30 minutes and is intended to provide patients with the knowledge and confidence needed to participate fully in SDM. The preparatory consultation takes place before the clinical encounter where the treatment decision will be made by a patient and HCP (i.e. a GP or specialized nurse who can prescribe medication). All participating HCPs from included practices receive a one-day, interactive interprofessional training in SDM, including general instructions to increase SDM knowledge and role-play with actors and fellow HCPs to improve skills. Various conversations will be practiced, varying in type of patient and situation. Moreover, the training will provide detailed information on the PDA and instructions on its usage. The training will be offered by PatientPlus. Besides the training offered by PatientPlus, the HCPs from included practices will follow a series of e-learnings that focus on the core of SDM. The e-learnings consist of realistic conversation recordings with reflection questions. They aim to provide the HCP insights into what SDM is and what it means for them in their daily work. The e-learnings are developed by ‘Programma Uitkomstgerichte Zorg’. Moreover, intervision meetings are held between the trained facilitators from all participating practices to stimulate interprofessional learning.

Patients and HCPs in the control practices will provide and receive usual care according to the national guidelines for T2DM of the Dutch College of General Practitioners (NHG). They will not have access to the multistage SDM program.

- 1. Use of co-intervention (if applicable)

Not applicable.

- 1. Escape medication (if applicable)

Not applicable.

1. INVESTIGATIONAL PRODUCT

Not applicable

- 1. Name and description of investigational product(s)
  2. Summary of findings from non-clinical studies
  3. Summary of findings from clinical studies
  4. Summary of known and potential risks and benefits
  5. Description and justification of route of administration and dosage
  6. Dosages, dosage modifications and method of administration
  7. Preparation and labelling of Investigational Medicinal Product
  8. Drug accountability

1. NON-INVESTIGATIONAL PRODUCT

Not applicable.

- 1. Name and description of non-investigational product(s)
  2. Summary of findings from non-clinical studies
  3. Summary of findings from clinical studies
  4. Summary of known and potential risks and benefits
  5. Description and justification of route of administration and dosage
  6. Dosages, dosage modifications and method of administration
  7. Preparation and labelling of Non Investigational Medicinal Product
  8. Drug accountability

1. METHODS
   1. Study parameters/endpoints
      1. Main study parameter/endpoint

The primary endpoints of the study are centered around three main aspects: 1) sample recruitment and retention; 2) study management; and 3) feasibility of outcome measurements. Each aspect has its own relevant outcome measures and measurement instruments.

Research question 1. Sample recruitment and retention

- - - - Recruitment, retention, and consent rates
      - Time required to recruit to the target
      - Sociodemographic (age, sex, country of birth, educational level, occupation, weight, length) and clinical characteristics (duration of T2DM and use of T2DM medication) of the T2DM participating patients

Research question 2. Study management

- - - - Assessment of primary care practices’ study management challenges, including time constraints and capacity issues.

Research question 3. Feasibility of outcome measurements

Assessment of understandability and accessibility of the measurement tools of the primary and secondary outcomes of the future economic evaluation. Additionally, we will collect data regarding the time required by patients to complete the questionnaires.

- - - - Primary outcomes:
        - Patient decisional conflict
        - Patient-rated level of SDM
        - Healthcare professionals rated level of SDM
        - Patient knowledge
      - Secondary outcomes
        - Medication adherence
        - Glycemic control (HbA1c)
        - Costs
        - Health-related quality of life
    1. Secondary study parameters/endpoints (if applicable)

Not applicable.

- - 1. Other study parameters (if applicable)

Not applicable.

- 1. Randomisation, blinding and treatment allocation

Our multistage SDM program will be piloted in a cluster-randomized controlled trial. Four primary care practices will be included in this pilot study. Randomization will be conducted by cluster (i.e. primary care practices) to avoid possible contamination between the intervention and control group [25]. Two primary care practices will be randomly assigned to the intervention group and two to the control group. Simple randomization will be used (by the researchers) to assign each primary care practice to a group with an equal probability (1:1 allocation) using a computerized random number generator [26]. Given the nature of the intervention, both patients with T2DM and HCPs are not blinded to the group assigned to them.

- 1. Study procedures

The pilot study will focus on three aspects: 1) sample recruitment and retention; 2) study management; and 3) feasibility of outcome measurements. Each aspect has its own relevant outcome measures and measurement instruments.

Research question 1. Sample recruitment and retention

To assess the extent to which a representative sample of T2DM patients is included and retained, quantitative data will be collected on: (1) recruitment, retention, and consent rates; (2) time required to recruit to target; and (3) sociodemographic and clinical characteristics. Sociodemographic and clinical characteristics include age, sex, country of birth, educational level, occupation, weight, length, duration of T2DM, and use of T2DM medication. To interpret the quantitative data and learn how to improve sample representativeness for the intended economic evaluation, additional qualitative data will be collected through one-hour focus groups with HCPs from the participating practices (one focus group per practice).

Research question 2. Study management

The focus groups with HCPs from participating practices will also be used to assess practices’ study management challenges. Through these focus groups, we aim to address key questions, such as whether primary care practices had adequate time to fulfill their committed tasks and if any capacity issues arose at the participating practices.

Research question 2. Feasibility of outcome measurements

We will assess the feasibility of several primary and secondary outcome measurements of the intended large-scale economic evaluation. The primary outcomes will focus on short-term SDM outcomes. The mid- and long-term cost-effectiveness of our multistage SDM program will serve as secondary outcomes.

Primary outcomes include patient decisional conflict, level of SDM as perceived by patients and HCPs, and patient knowledge. Decisional conflict is measured using the 16-item Decisional Conflict Scale for patients [37]. Patients will be asked to reflect on the treatment decision they made with their HCP and respond to 16 statements in the DCS using a five-point Likert scale (ranging from completely agree to completely disagree). Besides a total score, the DCS includes five dimensions (i.e. information, support, clarification or values, certainty, and decision quality) with higher scores on a five-point scale indicating more decisional conflict. Patient-rated level of SDM is measured by the 3-item CollaboRATE survey and the SDM-Q-9 questionnaire. The CollaboRATE survey assesses patients’ perception of being informed and engaged in the decision-making steps on a scale of zero (no effort was made) to nine (every effort was made) [38, 39]. The SDM-Q-9 questionnaire measures the extent of SDM during a consultation between the patient and HCP [40]. This questionnaire consists of nine statements each describing a different step of the SDM process. All items are scored on a six-point Likert scale from zero (completely disagree) to five (completely agree). The questionnaire also includes two open-ended questions on what health problem was the subject of the consultation and which decision was made. The SDM-Q-Doc questionnaire (SDM-Q-9 adapted to the HCP viewpoint) is used to measure the level of SDM during a consultation from the perspective of a HCP [40]. Patient knowledge is measured with nine tailor-made multiple-choice questions assessing patients’ understanding of the (risks and benefits of) glucose-lowering treatments.

Secondary outcomes include medication adherence, glycemic control, and societal costs. Medication adherence will be measured by the 5-item Medication Adherence Report Scale (MARS-5) and by collecting pharmacy records for diabetes medication [41]. MARS comprises five items that assess both intentional (not wanting to use it, changing doses, etc.) and non-intentional non-adherence (forgetting to take a dose). Respondents will be asked to rate the frequency of these events occurring on a 5-point Likert scale (ranging from never to always). Glycemic control will be assessed by obtaining HbA1c data from the general practitioner. Assessing costs and health-related quality of life is essential for conducting an economic evaluation since it allows us to compare the multistage program with usual care (in the intended economic evaluation) by estimating how much it costs to reach improvements in individuals’ quality of life [42]. A 9-item cost questionnaire will be used to measure relevant (non-)healthcare use as well as costs important to patients and families [43]. The Dutch EuroQol (EQ) 5D-5L questionnaire will be used to assess health-related quality of life [44]. This measure facilitates utility calculations and includes the EQ-5D dimension and the EQ visual analog scale (EQ VAS). EQ-5D comprises five dimensions: mobility, self-care, usual activities, pain, and anxiety. Each dimension is scored on a five-point Likert score (from no problems to extreme problems). The EQ VAS is used to assess the patient’s self-reported health on a visual analog scale. Table 1 provides an overview of the primary and secondary outcomes of the large-scale economic evaluation, including their measurement tools.

Semi-structured interviews with patients will be held to gain insight into the understandability and accessibility of the measurement tools. The interviews are optional for patients. When signing the informed consent, they have the option to indicate whether they grant permission for researchers to contact them for an interview. Interviews will be conducted either in person or online via Microsoft Teams, depending on the preference of the patient. Additionally, we will collect data regarding the time required by patients to complete the questionnaires and assess the prevalence of missing responses in the completed questionnaires. The focus groups with HCPs from participating practices will also be used to evaluate the understandability of the SDM-Q-Doc questionnaire.

**Table 1.** Outcomes of the intended trial-based economic evaluation, including their measurement tools. HCP: healthcare professional; SDM: shared decision-making.

| **Outcome** | **Assessed from** | **Measurement tool** | **Components** |
| --- | --- | --- | --- |
| Decisional Conflict | Patient | Decisional Conflict Scale (DCS) | Patients will be asked to reflect on the treatment decision they made with their HCP and respond to 16 statements in the DCS using a five-point Likert scale (from completely agree to completely disagree). |
| Patient-rated level of SDM | Patient | CollaboRATE survey | 3 statements that assess patients’ perception of being informed and engaged in decision-making steps on a scale of zero (no effort was made) to nine (every effort was made) |
|  |  | SDM-Q-9 questionnaire | The questionnaire contains nine statements each describing a different step of the SDM process. All items are scored on a six-point Likert scale from zero (completely disagree) to five (completely agree). The questionnaire also includes two open-ended questions on what health problem was the subject of the consultation and which decision was made. |
| HCP-level of SDM | HCP | SDM-Q-Doc questionnaire | SDM-Q-9 questionnaire adapted to the HCP viewpoint to assess the extent of SDM during a consultation from the perspective of a HCP. |
| Patient knowledge | Patient | 9 tailor-made multiple-choice questions | Assessing patients’ understanding of the (risks and benefits of) glucose-lowering treatments. |
| Medication adherence | Patient | Medication Adherence Report Scale (MARS-5) | Questionnaire that assesses unintentional and intentional non-adherence (self-reported). Patients are asked to rate the frequency of events (i.e. forgetting a dose, changing a dose) on a 5-point Likert scale (ranging from never to always). |
|  |  | Pharmacy data | Patient’s pharmacy records for all diabetes medication will be collected. |
| Glycemic control | Patient |  | Glycemic control is assessed by obtaining HbA1c data from the general practitioner. |
| Costs |  | 9-item cost questionnaire | The cost questionnaire aims to measure relevant (non-)healthcare use as well as costs important to patients and families. |
| Health-related quality of life | Patient | Dutch EuroQol (EQ) 5D-5L (quality of life) | The EQ 5D-5L assesses quality of life and includes the EQ-5D dimension and the EQ visual analog scale (VAS). EQ-5D comprises five dimensions: mobility, self-care, usual activities, pain, and anxiety. Each dimension of the EQ-5D is scored on a five-point Likert score (from no problems to extreme problems). The EQ VAS is used to assess the patient’s self-reported health on a visual analog scale. |

*Timeline*

The schedule of study enrolment and assessment can be found in Table 2. There are some differences in the timeline between participating patients in the intervention practices and control practices.

- - - - Intervention practices

Baseline measurements will capture sociodemographic and clinical patient characteristics, patient knowledge, glycemic control, medication adherence, costs, and health-related quality of life. Patients complete the DCS, CollaboRATE survey, and SDM-Q-9 questionnaire directly following the clinical encounter where a treatment decision is made.

- - - - Control practices

Patients will complete all questionnaires (sociodemographic and clinical characteristics, patient knowledge, glycemic control, medication adherence, costs, and health-related quality of life) directly following the clinical encounter where a treatment decision is made.

For both study arms, HCPs complete the SDM-Q-Doc questionnaire directly following the clinical encounter where a treatment decision is made. Follow-ups at 3 months and 9 months will facilitate the measurement of glycemic control, medication adherence, costs, and health-related quality of life. Semi-structured interviews with patients will be held within 1 month after study participation and the focus groups with HCPs from participating practices will be held at the end of the 9 months of implementation.

**Table 1.** Schedule of study enrolment and assessment.

|  |  | Enrolment |  |  |  |  |
| --- | --- | --- | --- | --- | --- | --- |
| Time point |  |  | t0 | t1 | t2 | t3 |
| Enrolment | Eligibility | x |  |  |  |  |
|  | Informed consent | x |  |  |  |  |
| Patient assessment (intervention) | Sociodemographic and clinical characteristics |  | x |  |  |  |
|  | Decisional Conflict |  |  | x | x | x |
|  | Patient-rated level of SDM |  |  | x | x | x |
|  | Patient knowledge |  | x |  |  |  |
|  | Medication adherence |  | x |  | x | x |
|  | Glycemic control |  | x |  | x | x |
|  | Costs |  | x |  | x | x |
|  | Health-related quality of life |  | x |  | x | x |
|  | Semi-structured interviews with patients |  |  | x |  |  |
| Patient assessment (control) | Sociodemographic and clinical characteristics |  |  | x |  |  |
|  | Decisional Conflict |  |  | x | x | x |
|  | Patient-rated level of SDM |  |  | x | x | x |
|  | Patient knowledge |  |  | x |  |  |
|  | Medication adherence |  |  | x | x | x |
|  | Glycemic control |  |  | x | x | x |
|  | Costs |  |  | x | x | x |
|  | Health-related quality of life |  |  | x | x | x |
|  | Semi-structured interviews with patients |  |  | x |  |  |
| HCP assessment | HCP-level of SDM |  |  | x |  |  |
|  | Focus group with HCPs |  |  |  |  | x |

t0 = baseline measurement; t1 = directly following the clinical encounter where a treatment decision is made; t2 = 3-month follow-up; t3 = 9-month follow-up. SDM: shared decision-making, HCP: healthcare professional.

All questionnaires (added as separate documents to the research protocol) will be included in the digital platform of PatientPlus. Participating patients and HCPs from the intervention practices will have an account giving them access to the PatientPlus platform. Patients and HCPs from the intervention practices are asked to complete the questionnaires via the online platform of PatientPlus. After 3 and 9 months, the patient will receive automatic notifications (via mail) prompting them to complete the follow-up questionnaires. Additionally, automated reminders will be sent if the patient has not yet completed the questionnaire.

Patients and HCPs from the control group will not have an account on the PatientPlus platform to avoid bias. They will complete questionnaires via Qualtrics [45]. After 3 and 9 months, the patient will receive automatic notifications (via mail) prompting them to complete the follow-up questionnaires. Additionally, automated reminders will be sent if the patient has not yet completed the questionnaire.

- 1. Withdrawal of individual subjects

Subjects can leave the study at any time for any reason if they wish to do so without any consequences. The investigator can decide to withdraw a subject from the study for urgent medical reasons.

- - 1. Specific criteria for withdrawal (if applicable)

Not applicable.

- 1. Replacement of individual subjects after withdrawal

Not applicable.

- 1. Follow-up of subjects withdrawn from treatment

Not applicable.

- 1. Premature termination of the study

Not applicable.

1. SAFETY REPORTING
   1. Temporary halt for reasons of subject safety

In accordance to section 10, subsection 4, of the WMO, the sponsor will suspend the study if there is sufficient ground that continuation of the study will jeopardise subject health or safety. The sponsor will notify the accredited METC without undue delay of a temporary halt including the reason for such an action. The study will be suspended pending a further positive decision by the accredited METC. The investigator will take care that all subjects are kept informed.

- 1. AEs, SAEs and SUSARs

The multistage SDM program will be implemented in two primary care practices in this pilot study. Although this is an intervention study, the intervention does not require the administration of any therapeutic agent. The PDA, as part of the multistage program, explicitly does not provide advice for a particular treatment. It aims to help patients with T2DM and HCPs to decide on the most suitable treatment. The direction for medication usage is at the discretion of a HCP in accordance with usual medical practice.

- - 1. Adverse events (AEs)

Adverse events are defined as any undesirable experience occurring to a subject during the study, whether or not considered related to the multistage SDM program for T2DM. Although an AE related to the multistage SDM program is not expected, all adverse events reported spontaneously by the subject or observed by the investigator or his staff will be recorded.

- - 1. Serious adverse events (SAEs)

A serious adverse event is any untoward medical occurrence or effect that

- results in death;
- is life threatening (at the time of the event);
- requires hospitalisation or prolongation of existing inpatients’ hospitalisation;
- results in persistent or significant disability or incapacity;
- is a congenital anomaly or birth defect; or
- any other important medical event that did not result in any of the outcomes listed above due to medical or surgical intervention but could have been based upon appropriate judgement by the investigator.

An elective hospital admission will not be considered as a serious adverse event.

The investigator must evaluate each SAE for causality and record causality for each event reported. The investigator will report all SAEs to the sponsor without undue delay after obtaining knowledge of the events.

The sponsor will report the SAEs through the web portal *ToetsingOnline* to the accredited METC that approved the protocol, within 7 days of first knowledge for SAEs that result in death or are life threatening followed by a period of maximum of 8 days to complete the initial preliminary report. All other SAEs will be reported within a period of maximum 15 days after the sponsor has first knowledge of the serious adverse events.

- - 1. Suspected unexpected serious adverse reactions (SUSARs)

Not applicable.

- 1. Annual safety report

Not applicable.

- 1. Follow-up of adverse events

All AEs will be followed until they have abated, or until a stable situation has been reached. Depending on the event, follow up may require additional tests or medical procedures as indicated, and/or referral to the general physician or a medical specialist.

SAEs need to be reported till end of study within the Netherlands, as defined in the protocol.

- 1. Data Safety Monitoring Board (DSMB) / Safety Committee

Not applicable.

1. STATISTICAL ANALYSIS
   1. Primary study parameter(s)

No substitution of missing or incomplete data will be done. The extent of missing data will be assessed, and reasons for missingness will be explored. Missing data patterns will be described to identify potential biases. Reporting and discussion of missing data will be included in the results to provide transparency and enable assessment of potential biases in the study.

Descriptive analysis will be conducted for quantitative data. Counts and percentages for non-continuous data and mean scores and standard deviations for continuous data will be calculated. This includes sociodemographic and clinical characteristics, primary outcomes, secondary outcomes, completion times of questionnaires, the prevalence of missing responses in the questionnaires, and estimates related to feasibility (e.g. recruitment rates). Descriptive analysis will be performed in Rstudio [46]. Descriptive statistics will be reported separately for each study aspect. No statistical analyses will be performed to determine the (cost-)effectiveness of the multistage SDM program since this is not the primary objective of the pilot study.

As to qualitative data, all interviews and focus groups will be audio-recorded and transcribed ad verbatim. Transcripts are analyzed in ATLAS.ti using thematic analysis with an inductive approach following three steps [47, 48]. First, the transcripts will be read and re-read in a process called ‘familiarization’. Second, phrases, sentences, and paragraphs with meaningful topics will be isolated and labeled by a code for each interview transcript. Two researchers will independently code part of the transcripts and compare and discuss codes for reflexivity, after which the remaining transcripts will be divided. Inter-coder reliability will be established and maintained throughout the coding process. Third, themes are developed by clustering codes with similar meanings or interrelations, to understand, interpret, and report the main insights flowing from the data. The insights gained by conducting interviews with patients with T2DM will be used to improve, where necessary, the understandability of the questionnaires used.

The data management process (i.e. data collection, data processing, data quality, and data analysis) and possible improvements thereof will be discussed with the research team in three 2-hour meetings during and after the completion of data analysis.

- 1. Secondary study parameter(s)

Not applicable

- 1. Other study parameters

Not applicable

- 1. Interim analysis (if applicable)

Not applicable

1. ETHICAL CONSIDERATIONS
   1. Regulation statement

The study will be conducted in agreement with the ethical standards described in the Declaration of Helsinki (October 2013) and in accordance with the Medical Research Involving Human Subjects Act (WMO).

- 1. Recruitment and consent

To recruit practices and patients, we collaborate with the primary care group ‘Huisarts & Zorg’, a provider network of 75 general practices in the region of Gorinchem (a municipality in South Holland). Four primary care practices from care group ‘Huisarts & Zorg’ will be included in this pilot study. Healthcare professionals from participating primary care practices will be asked to recruit adult patients with T2DM. Potentially eligible patients who are willing to learn more about the study receive a detailed study explanation (PIF, patiënteninformatie) on paper or via mail (depending on what the patient prefers) from their healthcare professional. Patients are provided with a clear explanation of the objectives and procedures of the study. They will be given the email address and telephone number of one of the researchers in case they have any questions. Most contacts will be made through their HCP to maintain trust and anonymity. An informed consent form will be attached to the PIF. If the patient decides to participate, they are asked to provide a signed version of the informed consent form to their healthcare professional before the start of the study. Informed consent is obtained before any study assessments are performed. The PIF has been drawn up using the patient information model (including declarations of consent and the subject information insurance text template) for subjects aged 16 years and older (adults). Participants can decide to opt out of the study at any time. The PIF and informed consent are attached to this protocol as separate documents.

- 1. Objection by minors or incapacitated subjects (if applicable)

Not applicable.

- 1. Benefits and risks assessment, group relatedness

The participation of patients with T2DM is crucial for the advancement of research in SDM and person-centered care. As described earlier, patients will receive detailed information about the study from their HCP. They will have sufficient time to make an informed decision on whether to participate. If a patient chooses not to participate, they will continue to receive usual care for T2DM. The inclusion of patients with T2DM in this pilot study is essential as their experiences and perspectives will significantly contribute to the refinement of trial methods for an economic evaluation, which eventually strengthens the limited evidence about the impact of person-centered care and SDM on mid- and long-term outcomes such as treatment adherence, health outcomes, and costs.

The primary focus of this study is to assess the feasibility of methods to improve the conduct of a future trial-based economic evaluation. Research methods must be both practical and effective while minimizing the burden and risk for patients with T2DM and healthcare professionals. Within this pilot study, we have carefully designed the protocol to ensure the burden and risks for patients are kept to a minimum. Participants will primarily be asked to complete non-invasive and relatively simple tasks, namely completing questionnaires. No medication interventions, treatments, or invasive procedures are involved, thereby minimizing physical strain and medical risks for the participants. We do not expect that there are any risks associated with completing the questionnaires. The questionnaires are based on validated measurement tools that have been widely used in research on similar health-related situations. They are designed to evaluate relevant aspects of treatment and care for T2DM, such as decisional conflict, the degree of SDM, and patient knowledge. Participants will not be asked where their answers are coming from, they do not have to talk about their experiences related to their care/disease, and they will not have to recall any stressful events that they might have had related to their condition.

We request that patients fill out questionnaires at three (control group) or four (intervention group) time points spanning 9 months: at the onset of participation, after 3 months, and at 9 months. These questionnaires are accessible through the PatientPlus online platform or Qualtrics (depending on whether the patient/HCP is in the intervention or control group). For the intervention group, the initial questionnaire comprises two parts, with part 1 being completed after signing the informed consent, taking approximately 5-10 minutes. Subsequently, upon making a treatment decision for type 2 diabetes with their healthcare provider, patients proceed to complete part 2, which requires around 15-25 minutes. Patients in the control group will complete part 1 and part 2 of the questionnaires at the same time (i.e. after the consult where a treatment decision was made). Automated reminders prompt patients to complete subsequent questionnaires after 3 and 9 months, each requiring approximately 5-10 minutes for completion.

Patient questionnaire responses will remain confidential and inaccessible to healthcare providers, ensuring the privacy of the patients. The interviews are optional for patients. When signing the informed consent, they have the option to indicate whether they grant permission for researchers to contact them for an interview. During the interviews, participants will be asked about the readability and understandability of the questionnaires. Thus, the study does not affect the mental integrity of the participants. Moreover, the study requires limited involvement of patients and therefore imposes no burden.

In summary, participating patients will have the opportunity to contribute to advancements in the field of person-centered care for the treatment of T2DM. The careful design of the study ensures the safety and well-being of the participants. The scientific and societal benefits of their involvement underscore the importance of this research in advancing the understanding and practice of person-centred care in the context of T2DM.

- 1. Compensation for injury

The sponsor has a liability insurance which is in accordance with article 7 of the WMO.

The sponsor (also) has an insurance which is in accordance with the legal requirements in the Netherlands (Article 7 WMO). This insurance provides cover for damage to research subjects through injury or death caused by the study.

The insurance applies to the damage that becomes apparent during the study or within 4 years after the end of the study.

- 1. Incentives (if applicable)

Not applicable.

1. ADMINISTRATIVE ASPECTS, MONITORING AND PUBLICATION
   1. Handling and storage of data and documents

The handling of personal data during the pilot study will comply with the EU General Data Protection Regulation and the Dutch Act on Implementation of the General Data Protection Regulation (in Dutch: Uitvoeringswet AVG, UAVG). Personal data will be processed and therefore the pilot study is recorded at the sponsor’s data processing register. Moreover, we will follow the FAIR principles, which are an integral part of Open Science, to improve the quality of the collected data. Data will be registered and archived at the end of the study, conforming to the FAIR principles. Data will be stored in the DataverseNL repository.

We will collect quantitative and qualitative data within the pilot study. We will collect qualitative data through focus groups with HCPs from the participating practices and semi-structured interviews with patients with T2DM. Interviews will be conducted either in person or online via Microsoft Teams, depending on the preference of the patient.

Quantitative data will be collected via PatientPlus and participating general practices from care group 'Huisarts & Zorg’. Our PDA for T2DM is included in the online catalog of PatientPlus. All questionnaires (added as separate documents to the research protocol) will be included in the digital platform of PatientPlus. Only patients and HCPs from the intervention practices will have access to the PDA via the PatientPlus platform. Patients and HCPs are asked to complete the questionnaires via the online platform of PatientPlus. Researchers of the Maastricht University will have an account for PatientPlus so they have access to the anonymised research data. PatientPlus only has access to the anonymised data. Participating HCPs do not have access to the completed questionnaires of the patients. PatientPlus stores the data in geographically separated data centers. CloudVPS is the data center provider. CloudVPS is both ISO 27001 and NEN 7510 certified. The CloudVPS network consists of three physically separated data centers. PatientPlus is also ISO27001 and NEN7510 certified since the beginning of 2020, meaning they have a properly functioning information security management system (ISMS). They guarantee the availability, integrity, and reliability of the health data they store. PatientPlus will also provide data on the usage behavior of patients for the decision aid (e.g. the duration of usage from start to end, time to complete questionnaires). Patients and HCPs from the control group will not have an account on the PatientPlus platform to avoid bias. They will complete questionnaires via Qualtrics [45]. The primary care practices will provide data on (1) recruitment, retention, and consent rates; (2) the time required to recruit to target; and 3) glycemic control (HbA1c values).

The data will be encrypted, with the key accessible only to the researchers directly involved in the study. Each participant interviewed or completing a questionnaire will be assigned a unique code (e.g. 1.1;1.2). Separate documents will be created for HCPs and patients who completed questionnaires: one including patient number, date of consultation, birth date, and a pseudonym, and another including the pseudonym with results of the questionnaires. We will store and backup the data on a secured network drive of the sponsor. The data will be stored for 10 years.

The following researchers will have access to the key:

- Dorijn Hertroijs
- Anna Tichler
- Arianne Elissen

Documentation of the data will be done to keep data usable and understandable. The data documentation will include information about the context of the data collection, collection methods, data manipulations, different versions, etc. A folder structure will be defined before the start of the data collection.

A data management plan was drawn and approved by the data steward of the sponsor. The data management plan is attached to the protocol as a separate document. The plan encompasses key elements such as data processing and data archiving.

- 1. Monitoring and Quality Assurance

Not applicable.

- 1. Amendments

Amendments are changes made to the research after a favourable opinion by the accredited METC has been given. All amendments will be notified to the METC that gave a favourable opinion.

- 1. Annual progress report

The investigator will submit a summary of the progress of the trial to the accredited METC once a year. Information will be provided on the date of inclusion of the first subject, numbers of subjects included and numbers of subjects that have completed the trial, serious adverse events/ serious adverse reactions, other problems, and amendments.

- 1. Temporary halt and (prematurely) end of study report

The investigator will notify the accredited METC of the end of the study within a period of 8 weeks. The end of the study is defined as the completion of the questionnaires at 9-month follow-up of the last patient.

The sponsor will notify the METC immediately of a temporary halt of the study, including the reason of such an action.

In case the study is ended prematurely, the sponsor will notify the accredited METC within 15 days, including the reasons for the premature termination.

Within one year after the end of the study, the investigator/sponsor will submit a final study report with the results of the study, including any publications/abstracts of the study, to the accredited METC.

- 1. Public disclosure and publication policy

Public disclosure and publication of research data will follow the ethical guidelines set forth by the Central Committee on Research Involving Human Subjects (CCMO), as outlined in the CCMO statement on Publication Policy (<https://www.ccmo.nl/publicaties/publicaties/2002/03/15/ccmo-notitie-publicatiebeleid>). Results of scientific research involving human subjects will be disclosed unreservedly. The results of this pilot study will be disseminated through conference presentations and international peer-reviewed scientific journals. Participating patients are informed in the PIF (proefpersoneninformatie) that research results will be made public. All publications resulting from scientific research that is fully or partially funded by the subsidizing party (ZonMw) will be made available immediately (without embargo) in Open Access, as determined in accordance with the ZonMw Open Access policy. Authorship shall be determined in accordance with the Uniform Requirements for Manuscripts submitted to Biomedical Journals (<http://www.icmje.org/>). All persons who are eligible to be an author on a publication must be listed as an author unless that person indicates that they do not wish to participate in a given publication. All eligible authors are responsible for reviewing and approving the manuscripts before submission. The subsidizing party will be acknowledged in publications. The sponsor (Maastricht University) performs independent academic research. They will take the lead in authoring a publication and shall confirm the wishes of each potential author for inclusion in a particular publication before its submission or dissemination. The sponsor will publish both positive and negative study results.

1. STRUCTURED RISK ANALYSIS

Not applicable.

- 1. Potential issues of concern
  2. Synthesis

1. REFERENCES

1. International Diabetes Federation. IDF Diabetes Atlas. Brussels, Belgium; 2021. Available at: https://www.diabetesatlas.org.

2. Elwyn G, O'Connor A, Stacey D, Volk R, Edwards A, Coulter A, et al. Developing a quality criteria framework for patient decision aids: online international Delphi consensus process. BMJ (Clinical research ed). 2006;333(7565):417-.

3. ElSayed NA, Aleppo G, Aroda VR, Bannuru RR, Brown FM, Bruemmer D, et al. 9. Pharmacologic Approaches to Glycemic Treatment: Standards of Care in Diabetes—2023. Diabetes Care. 2022;46(Supplement_1):S140-S57.

4. Davies MJ, Aroda VR, Collins BS, Gabbay RA, Green J, Maruthur NM, et al. Management of hyperglycaemia in type 2 diabetes, 2022. A consensus report by the American Diabetes Association (ADA) and the European Association for the Study of Diabetes (EASD). Diabetologia. 2022.

5. Nederlands Huisartsen Genootschap. NHG-standaard Diabetes mellitus type 2 (versie 5.6). 2023.

6. Institute of Medicine. Crossing the Quality Chasm: A New Health System for the 21st Century. Washington (DC): National Academies Press (US); 2001. p. 360.

7. Stacey D, Legare F, Col NF, Bennett CL, Barry MJ, Eden KB, et al. Decision aids for people facing health treatment or screening decisions. Cochrane Database Syst Rev. 2014(1):CD001431.

8. Stacey D, Suwalska V, Boland L, Lewis KB, Presseau J, Thomson R. Are Patient Decision Aids Used in Clinical Practice after Rigorous Evaluation? A Survey of Trial Authors. Med Decis Making. 2019;39(7):805-15.

9. Kellar I, Mann E, Kinmonth AL, Prevost AT, Sutton S, Marteau TM. Can informed choice invitations lead to inequities in intentions to make lifestyle changes among participants in a primary care diabetes screening programme? Evidence from a randomized trial. Public Health. 2011;125(9):645-52.

10. Branda ME, LeBlanc A, Shah ND, Tiedje K, Ruud K, Van Houten H, et al. Shared decision making for patients with type 2 diabetes: a randomized trial in primary care. BMC Health Serv Res. 2013;13:301-.

11. Mathers N, Ng CJ, Campbell MJ, Colwell B, Brown I, Bradley A. Clinical effectiveness of a patient decision aid to improve decision quality and glycaemic control in people with diabetes making treatment choices: a cluster randomised controlled trial (PANDAs) in general practice. BMJ Open. 2012;2(6):e001469.

12. Mullan RJ, Montori VM, Shah ND, Christianson TJH, Bryant SC, Guyatt GH, et al. The Diabetes Mellitus Medication Choice Decision Aid: A Randomized Trial. Arch Intern Med. 2009;169(17):1560-8.

13. Thabane L, Ma J, Chu R, Cheng J, Ismaila A, Rios LP, et al. A tutorial on pilot studies: the what, why and how. BMC Med Res Methodol. 2010;10(1):1.

14. Van Teijlingen E, Hundley V. The importance of pilot studies. Social research update. 2001(35):1-4.

15. Tichler A, Hertroijs D, Ruwaard D, Brouwers M, Elissen A. Development of a patient decision aid for type 2 diabetes mellitus: a patient-centered approach. Research Square [Preprint] [posted 2023 Oct 10; cited 2023 Oct 11]. 2023.

16. Black SA. Diabetes, diversity, and disparity: what do we do with the evidence? Am J Public Health. 2002;92(4):543-8.

17. Brodaty H, Gibson LH, Waine ML, Shell AM, Lilian R, Pond CD. Research in general practice: a survey of incentives and disincentives for research participation. Ment Health Fam Med. 2013;10(3):163-73.

18. McKinley N, McCain RS, Convie L, Clarke M, Dempster M, Campbell WJ, Kirk SJ. Resilience, burnout and coping mechanisms in UK doctors: a cross-sectional study. BMJ Open. 2020;10(1):e031765.

19. West CP, Dyrbye LN, Shanafelt TD. Physician burnout: contributors, consequences and solutions. J Intern Med. 2018;283(6):516-29.

20. Willems AEM, Heijmans M, Brabers AEM, Rademakers J. Gezondheidsvaardigheden in Nederland: factsheet cijfers 2021. Utrecht: Nivel. 2022.

21. Chan A-W, Tetzlaff JM, Altman DG, Laupacis A, Gøtzsche PC, Krleža-Jerić K, et al. SPIRIT 2013 Statement: Defining Standard Protocol Items for Clinical Trials. Ann Intern Med. 2013;158(3):200-7.

22. Chan A-W, Tetzlaff JM, Gøtzsche PC, Altman DG, Mann H, Berlin JA, et al. SPIRIT 2013 explanation and elaboration: guidance for protocols of clinical trials. BMJ : British Medical Journal. 2013;346:e7586.

23. Husereau D, Drummond M, Augustovski F, de Bekker-Grob E, Briggs AH, Carswell C, et al. Consolidated Health Economic Evaluation Reporting Standards 2022 (CHEERS 2022) Statement: Updated Reporting Guidance for Health Economic Evaluations. Value Health. 2022;25(1):3-9.

24. Nederland Z. Richtlijn voor het uitvoeren van economische evaluaties in de gezondheidszorg. 2016. p. 38.

25. Fayers PM, Jordhùy MS, Kaasa S. Cluster-randomized trials. Palliat Med. 2002;16(1):69-70.

26. Kang M, Ragan BG, Park JH. Issues in outcomes research: an overview of randomization techniques for clinical trials. J Athl Train. 2008;43(2):215-21.

27. InEen. Benchmark Transparante Ketenzorg 2022. Rapportage zorgprogramma's diabetes mellitus type 2, COPD, Astma en Vasculair Risico Management bij patiënten met een hartvaatziekte en een verhoogd risico op hartvaatziekten. 2023.

28. Dierick-van Daele ATM, Metsemakers JFM, Derckx EWCC, Spreeuwenberg C, Vrijhoef HJM. Nurse practitioners substituting for general practitioners: randomized controlled trial. J Adv Nurs. 2009;65(2):391-401.

29. Whitehead AL, Julious SA, Cooper CL, Campbell MJ. Estimating the sample size for a pilot randomised trial to minimise the overall trial sample size for the external pilot and main trial for a continuous outcome variable. Stat Methods Med Res. 2016;25(3):1057-73.

30. Yu CH, Ivers NM, Stacey D, Rezmovitz J, Telner D, Thorpe K, et al. Impact of an interprofessional shared decision-making and goal-setting decision aid for patients with diabetes on decisional conflict – study protocol for a randomized controlled trial. Trials. 2015;16(1):286.

31. Tichler A, Hertroijs DFL, Ruwaard D, Brouwers M, Hiligsmann M, de Jong JD, Elissen AMJ. Preferred Conversation Topics with Respect to Treatment Decisions Among Individuals with Type 2 Diabetes. Patient Prefer Adherence. 2023;17:719-29.

32. Elwyn G, O'Connor AM, Bennett C, Newcombe RG, Politi M, Durand MA, et al. Assessing the quality of decision support technologies using the International Patient Decision Aid Standards instrument (IPDASi). PLoS One. 2009;4(3):e4705.

33. Boland L, McIsaac DI, Lawson ML. Barriers to and facilitators of implementing shared decision making and decision support in a paediatric hospital: A descriptive study. Paediatr Child Health. 2016;21(3):e17-21.

34. Elwyn G, Scholl I, Tietbohl C, Mann M, Edwards AGK, Clay C, et al. “Many miles to go …”: a systematic review of the implementation of patient decision support interventions into routine clinical practice. BMC Medical Informatics and Decision Making. 2013;13(2):S14.

35. Légaré F, Ratté S, Gravel K, Graham ID. Barriers and facilitators to implementing shared decision-making in clinical practice: Update of a systematic review of health professionals’ perceptions. Patient Educ Couns. 2008;73(3):526-35.

36. Joseph-Williams N, Edwards A, Elwyn G. Power imbalance prevents shared decision making. BMJ. 2014;348:g3178.

37. O'Connor AM. Validation of a Decisional Conflict Scale. Med Decis Making. 1995;15(1):25-30.

38. Barr PJ, Thompson R, Walsh T, Grande SW, Ozanne EM, Elwyn G. The psychometric properties of CollaboRATE: a fast and frugal patient-reported measure of the shared decision-making process. J Med Internet Res. 2014;16(1):e2-e.

39. Elwyn G, Barr PJ, Grande SW, Thompson R, Walsh T, Ozanne EM. Developing CollaboRATE: a fast and frugal patient-reported measure of shared decision making in clinical encounters. Patient Educ Couns. 2013;93(1):102-7.

40. Rodenburg-Vandenbussche S, Pieterse AH, Kroonenberg PM, Scholl I, van der Weijden T, Luyten GP, et al. Dutch Translation and Psychometric Testing of the 9-Item Shared Decision Making Questionnaire (SDM-Q-9) and Shared Decision Making Questionnaire-Physician Version (SDM-Q-Doc) in Primary and Secondary Care. PLoS One. 2015;10(7):e0132158.

41. Chan AHY, Horne R, Hankins M, Chisari C. The Medication Adherence Report Scale: A measurement tool for eliciting patients' reports of nonadherence. Br J Clin Pharmacol. 2020;86(7):1281-8.

42. Fox-Rushby J. CJ. Economic Evaluation. London: Oxford University Press; 2006.

43. Janssen LMM, Hiligsmann M, Elissen AMJ, Joore MA, Schaper NC, Bosma JHA, et al. Burden of disease of type 2 diabetes mellitus: cost of illness and quality of life estimated using the Maastricht Study. Diabet Med. 2020;37(10):1759-65.

44. EuroQol. EuroQol 2017 [10-10-2023]. Available from: <https://euroqol.org/eq-5d->instruments/eq-5d-3l-about/.

45. Qualtrics. Provo, Utah, USA, 2005 [Available from: https://www.qualtrics.com/.

46. Team R. RStudio: Integrated Development for R. 1.4.1106 ed. Boston, MA: RStudio, PBC; 2020.

47. Braun V, Clarke V. Using thematic analysis in psychology. Qualitative Research in Psychology. 2006;3(2):77-101.

48. ATLAS.ti Scientific Software Development GmbH. ATLAS.ti Scientific Software Development GmbH.
